# Efficient Synthesis of Various Substituted (Thio)Ureas, Semicarbazides, Thiosemicarbazides, Thiazolidones, and Oxadiazole Derived from [2.2]Paracyclophane

**DOI:** 10.1021/acsomega.2c00141

**Published:** 2022-04-06

**Authors:** Mohammed B. Alshammari, Ashraf A. Aly, Stefan Bräse, Martin Nieger, Lamiaa E. Abd El-Haleem

**Affiliations:** † Chemistry Department, College of Sciences and Humanities, Prince Sattam bin Abdulaziz University, P.O. 10, Box 83, Al-Kharij 11942, Saudi Arabia; ‡ Chemistry Department, Faculty of Science, Minia University, 61519 El-Minia, Egypt; § Institute of Organic Chemistry, Karlsruhe Institute of Technology, 76131 Karlsruhe, Germany; ∥ Institute of Biological and Chemical Systems (IBCS-FMS), Karlsruhe Institute of Technology, 76344 Eggenstein-Leopoldshafen, Germany; ⊥ Department of Chemistry, University of Helsinki, P.O. Box 55 (A. I. Virtasen Aukio I), 00014 Helsinki, Finland

## Abstract

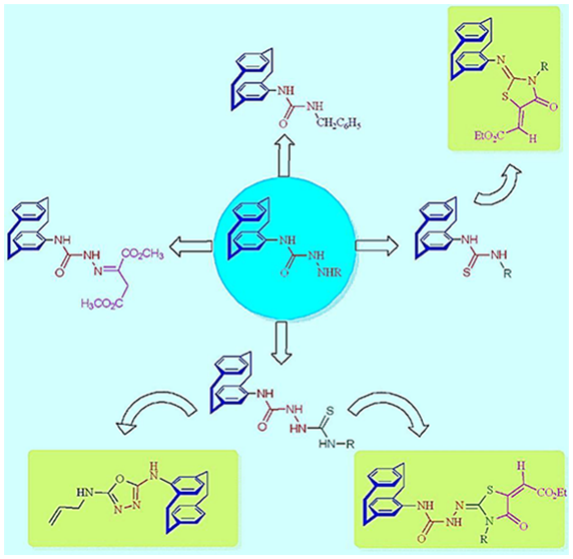

The strategies of the syntheses of various (thio)ureas, semicarbazides, thiosemicarbazides, thiazolidones, and oxadiazole derived from the [2.2]paracyclophane molecule are achieved starting with 4-(2.2]paracyclophanyl)isocyanate. The structures of the obtained products were elucidated by NMR, mass spectrometry, and infrared (IR) spectroscopy in addition to high-resolution mass spectrometry (HRMS). X-ray structure analysis was also used to prove the assigned structure.

## 1 Introduction

[2.2]Paracyclophane (PC) chemistry has evolved from the functional molecules to functional materials and from the synthetic curiosity to emerging applications in asymmetric synthesis, energy materials, π-stacked polymers, and functional parylene coatings (i.e. polymer made by polymerization of PC induced by vapor-phase pyrolysis).^1−4^ [2.2]Paracyclophane is also described as a rigid molecule within the interior of the conjugated segment with an otherwise similar aspect ratio to the phenylene unit. The intermolecular interactions in PC involving aromatic rings are the key processes in both chemical and biological recognition.^5^

**Figure 1:**
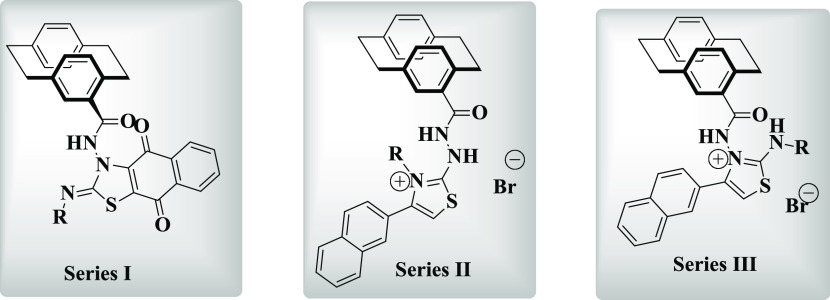
Different series of paracyclophanyl-thiazole derivatives (**I−III**) as anticancer agents.

Recently, it has been shown that connecting heterocycles with the PC moiety showed anticancer activity as in the case of paracyclophanyl-dihydronaphtho[2,3-*d*]thiazoles and paracyclophanyl-thiazolium bromides.^6^ Among the following three assigned series **I**−**III** of the synthesized paracyclophanyl-heterocycles (Figure 1), series I having 1,4-dihydronaphthoquinone, was found as more active as antiproliferative agents than their naphthalene-containing congeners (series II and III) toward the SK-MEL-5 melanoma cell line.^6^

Previously, we reported the various classes of connection between PC and heterocycle moieties.^7^ Aly et al. synthesized heterocycles conjugated to [2.2]paracyclophane such as five-membered rings (i.e., imidazolinone,^8^ pyrrole,^9^ triazolethiones, and substituted oxadiazoles^10^) together with six-membered rings (i.e., pyridine).^11,12^

It was reported that some marketed drugs had been found to contain the *N*-acylhydrazone motif in their structures, e.g., azumolene, carbazochrome, dantrolene, nitrofurantoin, nitrofurazone, nifuroxazide, and testosterone 17-enanthate 3-benzilic acid hydrazone.^13^ More specifically, acylhydrazide-based compounds have shown antioxidant activities.^14^ Hydrazides and carbohydrazides have been described as useful building blocks for the assembly of various heterocyclic rings.^15−19^ Ureas and thioureas in combination with benzothiazoles were reported that they produced DNA topoisomerase or HIV reverse transcriptase inhibitors.^20−22^ 1,3,4-Oxadiazole heterocyclic ring is one of the most important heterocyclic moieties due to its versatile biological actions.^23^ Based upon the aforementioned, we are encouraged to incorporate a PC molecule to (thio)urea, semicarbazides, thiosemicarbazide, thiazolidone, and oxadiazole groups.

## 2 Results and Discussion

### 2.1 Synthesis of 1*N*-Benzyl-3-*N*-[2.2]paracyclophanylurea (**6**) and *N*-(4′-[2.2]Paracyclo-phanyl)hydrazinecarboxamides **7a**, **7b**

**Scheme 1:**
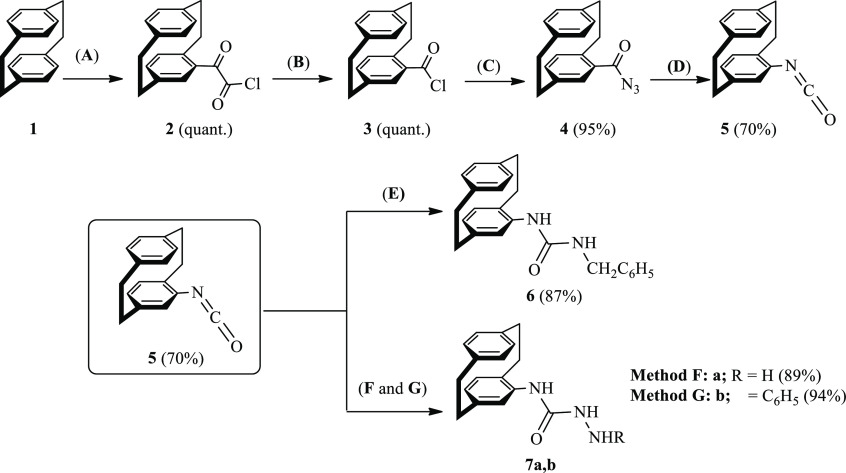
Synthesis of 1*N*-Benzyl-3-*N*-[2.2]paracyclophanylurea (**6**) and *N*-(4′-[2.2]Paracyclophanyl)hydrazinecarboxamides **7a** and **7b** Reagents and conditions: (A) (COCl)_2_/AlCl_3_, −10 to 5 °C, 20 min; (B) PhCl, Δ, 40 h; (C) NaN_3_, acetone/water, r.t., 2 h; (D) toluene, 80 °C, 1 h; (E) PhCH_2_NH_2_/fusion, 100 °C, 10 h; (F) NH_2_NH_2_ as a solvent, Δ, 20 h; (G) PhNHNH_2_, toluene, 20 h.

**Scheme 2:**
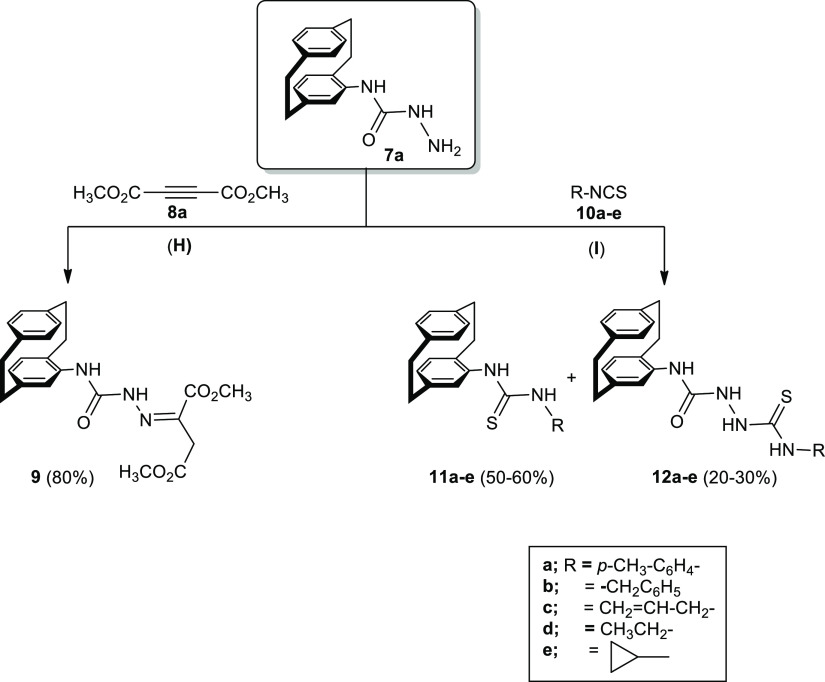
Strategy of Various Reactions of *N*-(4′-[2.2]Paracyclophanyl)hydrazinecarboxamide (**7a**) Reagents and conditions: (H) EtOH, reflux 4 h; (I) oil path EtOH, 70 °C, reflux 4–8 h.

**Scheme 3:**
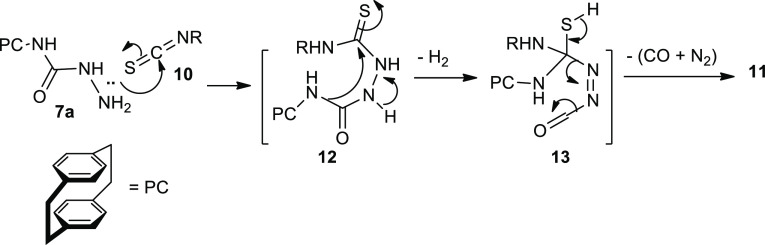
Mechanism Describing the Formation of Compounds **11a**–**11e** and **12a**–**12e**

**Scheme 4:**
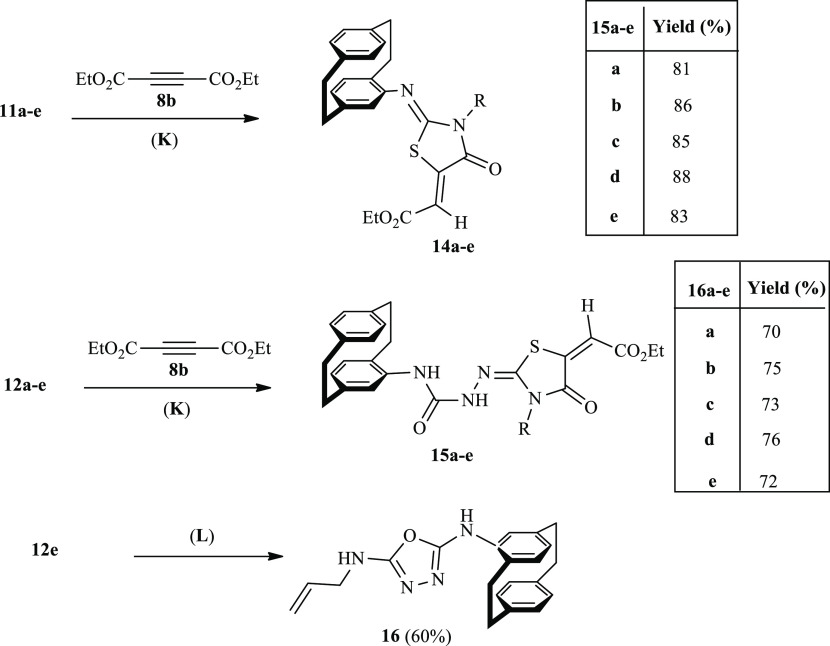
Synthesis of Thiazoles **14a**–**14e** and **15a**–**15e** in Addition to 1,3,4-Oxadiazole Derivative **16** Reagents and conditions: (K) EtOH, reflux; (L) NaOH (2 N), EtOH, reflux 3 h.

The strategy of preparing compounds **6**, **7a**, and **7b** was divided into two parts: First, starting with the parent hydrocarbon **1** as a commercial product, which was then converted into the acid chloride derivative **3**(24) by the procedure described in Scheme 1. At the beginning, compound **1** was converted into **2** during reaction with oxalyl chloride/aluminum trichloride. Then, heating **2** in refluxing chlorobenzene caused decarbonylation to give **3**. Subsequently, the resulting acid chloride **3** was subjected toward NaN_3_/acetone to give compound **4**(24) (Scheme 1). Heating **4** in toluene at 80 °C provided the corresponding isothionate **5**(24) in 70% yield (Scheme 1). Second, fusion of **5** with benzylamine gave the corresponding urea **6** in 87% yield (Scheme 1). Based on NMR, IR, mass spectra, as well as HRMS, the structure of compound **6** was satisfactorily proved. As the ^1^H NMR spectrum indicated the appearance of the CH_2_ protons of compound **6** as a doublet at δ_H_ = 4.26 (*J* = 6.0 Hz). Whereas, the two NH protons appeared as two singlets at δ_H_ = 7.73 and 6.75 ppm. In ^13^C NMR, the CH_2_ and the carbonyl carbon signals resonated at δ_C_ = 42.9 and 158.1 ppm, respectively. On subjecting **5** with hydrazines by the procedure mentioned in Scheme 1, *N*-(4′-[2.2]paracyclophanyl)hydrazinecarboxamides **7a** and **7b** were obtained in very good yields (Scheme 1). The structure of the newly prepared compound **7a** was established by IR, NMR, mass spectra, as well as HRMS. The IR spectrum revealed a diagnostic broad band at *ṽ* = 3352–3214 for NH groups, whereas the carbonyl group appeared at *ṽ* = 1632 cm^–1^. The ^1^H NMR spectrum exhibited the NH-2 and NH-1 protons at δ_H_ = 7.59 and 6.88 ppm, respectively. In addition, the characteristic hydrazine-NH_2_ resonated in the ^1^H NMR spectrum at δ_H_ = 4.72 ppm. The ^13^C NMR spectrum displayed the carbonyl-carbon at δ_C_ = 157.3, whereas the four distinctive CH_2_-bridged carbons of PC resonated at δ_C_ = 35.4, 35.1, 32.9, and 32.3 ppm. HRMS proved the chemical formula of **7a** as C_17_H_19_N_3_O.

For compound **7b**, HRMS confirmed the molecular formula of compound **7b** as C_23_H_23_N_3_O. The ^1^H NMR spectrum revealed the NH protons as three singlets at δ_H_ = 8.36 (for NH-2), 7.97 (for NH-1), and 6.60 ppm for (NH-3). The ^13^C NMR spectrum of compound **7b** revealed the carbonyl carbon at δ_C_ = 155.8, whereas the carbon signal of C-Ph was observed at δ_C_ = 149.1 ppm (see the Experimental Section). The four carbon signals of the CH_2_–CH_2_ appeared at δ_C_ = 36.4, 36.1, 35.7, and 32.2 ppm.

### 2.2 Reaction of Compound **7a** with Dimethyl Acetylenedicarboxylate (**8a**) and Substituted Isothiocyanates **10a**–**10e**

In extension to the aforesaid strategy and taking compound **7a**, as an example, in the reaction between **7a** and dimethyl acetylenedicarboxylate (**8a**), the reaction gave compound **9** in 80% yield (Scheme 2). HRMS confirmed the molecular formula of **9** as C_23_H_25_N_3_O_5_ indicating the addition reaction of compound **7a** to **8a** proceeded without elimination of a MeOH molecule.

**Figure 2:**
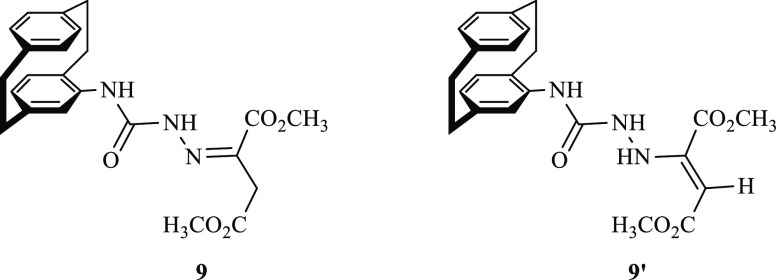
Additive products **9** and **9′** from the reaction between **7a** and **8a**.

**Figure 3:**
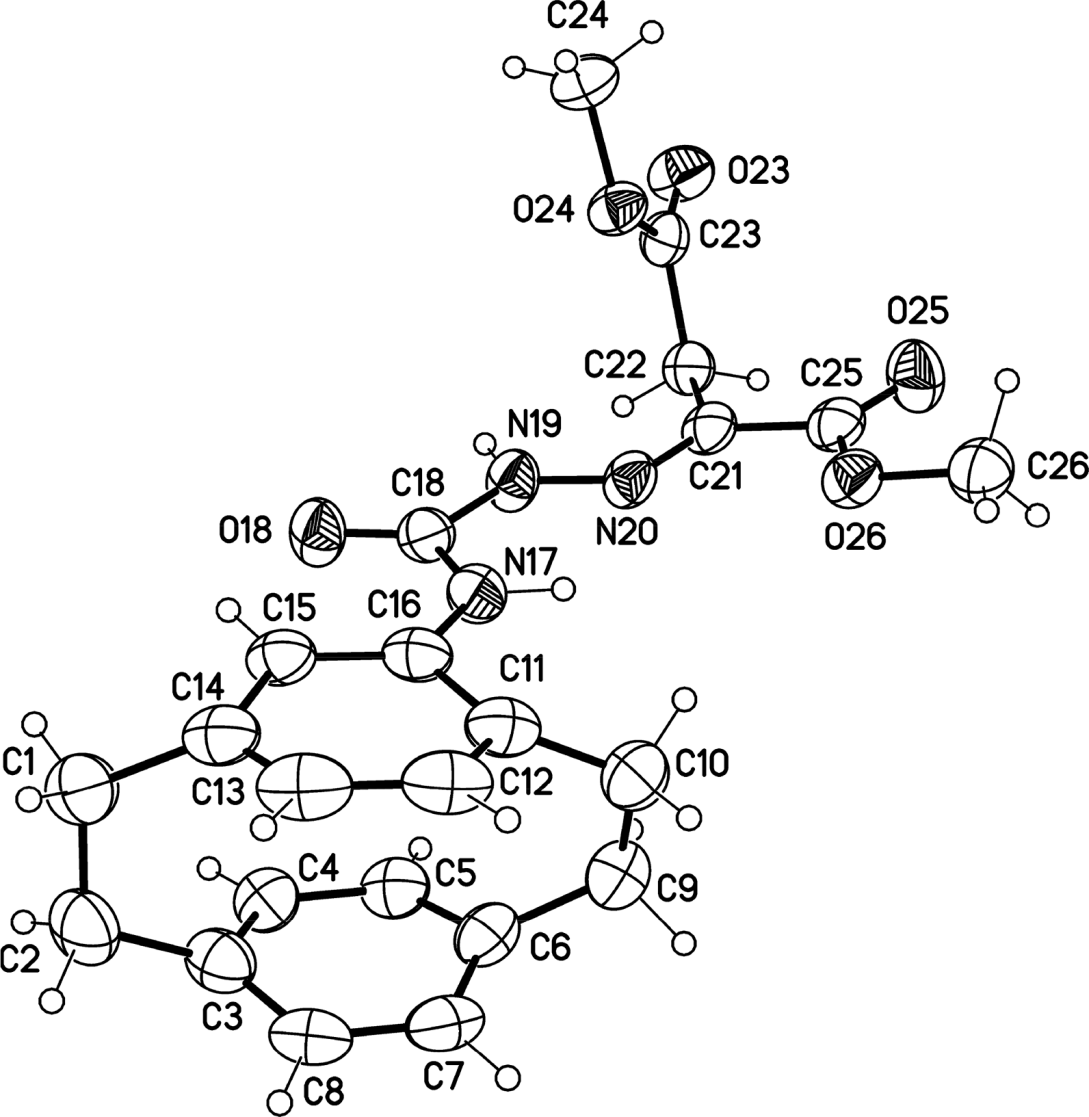
Molecular structure of compound **9** (displacement parameters are drawn at the 50% probability level).

To discriminate between the possible structures **9** and **9′**, we analyzed the NMR spectrum. As, the hydrazano-NH appeared in the ^1^H NMR spectrum as a singlet at δ_H_ = 11.01, whereas the PC-NH at δ_H_ = 8.45. The two methyl-ester protons appeared as two very close singlets at δ_H_ = 3.90 and 3.75 ppm. The ^1^H NMR did not reveal any proton for the ethylenic-H, which excluded the formation of the isomeric product **9′** (Figure 2). The CH_2_ carbon and its protons attached to the ester group resonated at the same region of the ethylenic-CH_2_ of PC. The ^13^C NMR spectrum revealed the two methyl-esters at δ_C_ = 52.5 and 52.1 ppm (see the Experimental Section). The structure of **9** was unambiguously proved by X-ray structure analysis as shown in Figure 3.

X-ray structure analysis of compound **9** showed different bond lengths of the C–N bonds, as the bond lengths of C16–N17 and C18–N19 are 1.413 and 1.384 Å, respectively. The lengths of the double bonds assigned to the C=O and N=C as in C18–O18 and N20–C21 are 1.225 and 1.272 Å, respectively. Whereas the lengths of the C–C bond assigned to the C21–C22 and C22–C23 are 1.496 and 1.510 Å, respectively.

**Figure 4:**
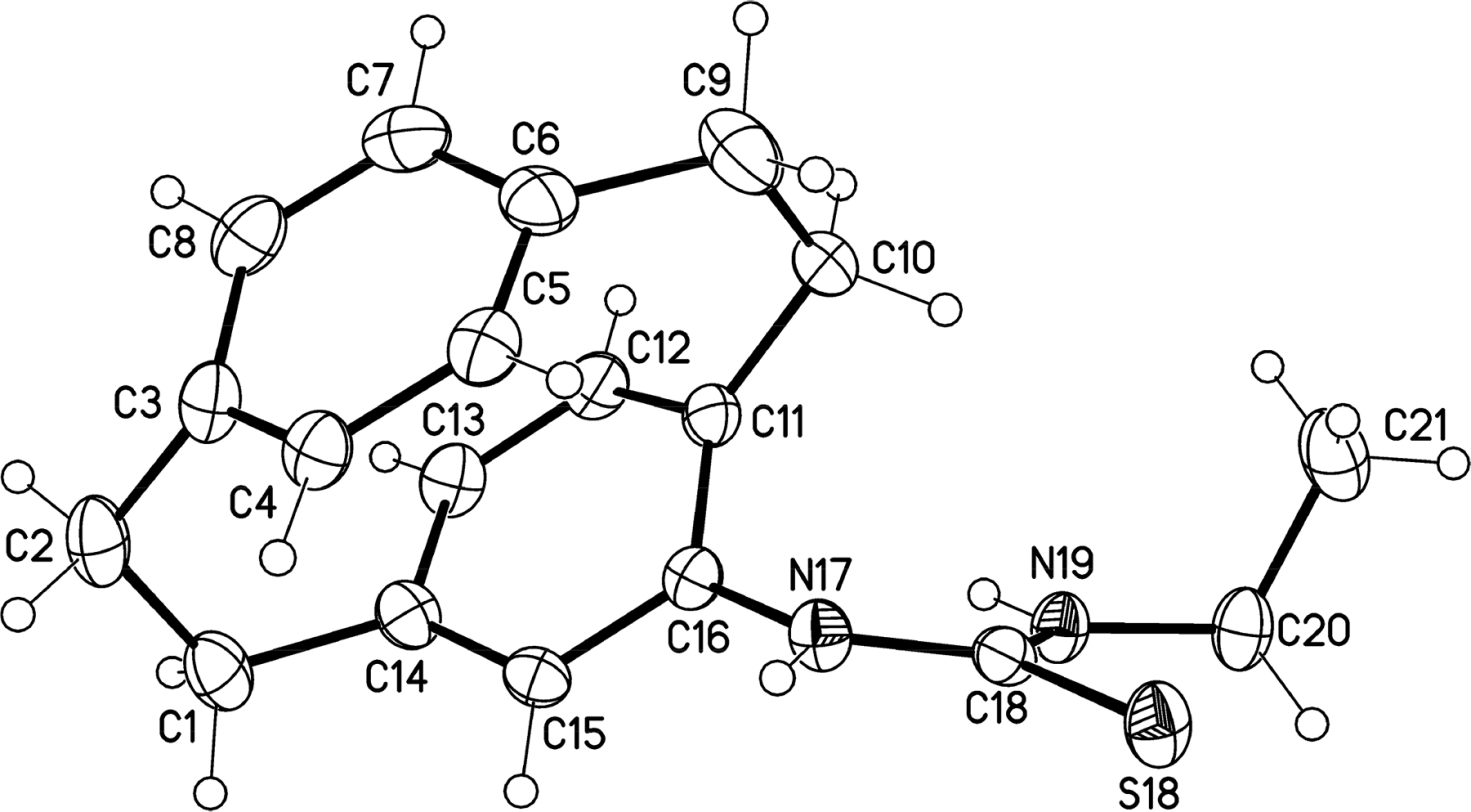
Molecular structure of one of the crystallographic independent molecules of compound **11d** (displacement parameters are drawn at the 50% probability level).

Surprisingly, when compound **7a** was subjected to substituted isothiocyanates **10a**–**10e**, the unexpected substituted thiourea derivatives **11a**–**11e** were obtained in 50–60% yields as the major products, whereas the expected products results in the addition reaction of **7a** to **10a**–**10e** were obtained in 20–30% yields (Scheme 2). Both products were separated by column chromatography using ethyl acetate–hexane, 10:1. The IR spectrum of compound **11d**, as an example, revealed absorptions at *ṽ* = 3296–3206 (NH, s), 3091 (aryl-H), 2925 (aliph.-CH), and 1456 cm^–1^ (C=S). Additionally, the ^1^H NMR spectrum revealed two singlets at δ_H_ = 8.99 (NH-1) and 7.50 ppm (NH-3). The ethyl protons were detected in the ^1^H NMR spectrum as a quartet at δ_H_ = 3.61 (for CH_2_, *J* = 7.2 Hz) and as a double-triplet at δ_H_ = 1.08 ppm (for CH_3_, *J* = 13.2, 7.1 Hz). The ^13^C NMR spectrum presented the C=S and the ethyl carbon signals at δ_C_ = 180.2, 56.5 (CH_2_-ethyl) and 14.9 ppm (CH_3_-ethyl), respectively. HRMS confirmed the molecular formula of **11d** as C_19_H_22_N_2_S. Finally, X-ray structure analysis confirmed the structure of compound **11d** as shown in Figure 4.

The structures of compounds **12a**–**12e** were identified as thiamido derivatives of **11a**–**11e** (Scheme 2). As for example, compound **12d** was proved as *N*-(4′-[2.2]paracyclophanyl)-2-(ethylcarbamothioyl)hydrazine-1-carboxamide. In the ^1^H NMR spectrum, compound **12d** supported the structure, since four singlets for NH protons appeared at δ_H_ = 9.29 (NH-3), 8.37 (NH-2), 8.29 (NH-4), and 7.59 ppm (NH-1). The ethyl protons resonated in the ^1^H NMR spectrum as a quartet at δ_*H*_ = 3.61 (CH_2_, *J* = 7.2 Hz) and as a triplet for CH_3_ at δ_H_ = 1.12 ppm (*J* = 7.1 Hz). The ^13^C NMR spectrum confirmed the structure of **12d** by the appearance of the C=S carbon signal at δ_C_ = 182.6, in addition to a signal at δ_C_ = 155.0 ppm for the carbonyl carbon signal. The ethyl carbon signals were distinguished at δ_C_ = 39.0 (CH_2_-ethyl) and at δ_C_ = 14.9 ppm (CH_3_-ethyl). Mass spectrometry showed the molecular ion peak at *m*/*z* (%) = 368 (20). Besides that, HRMS proved the molecular formula of **12d** to be C_20_H_24_N_3_OS.

The mechanism describes the formation of compounds **11a**–**11e** and **12a**–**12e** could be explained as due to the addition of the NH lone pair to the electrophilic center in **10a**–**10d** in the C=S to form compound **11** (Scheme 3). Rearrangement of **11** involved addition of the NH-PC *via* the bond between NH-PC and C=O to the electrophilic carbon of C=S accompanied by the oxidation process to give the intermediate **12** (Scheme 3). Upon heating, N_2_ and CO would then be eliminated, as shown in Scheme 3, to produce **11** (Scheme 3).

### 2.3 Reaction of Compounds **11a**–**11e** and **12a**–**12e** with Diethyl Acetylenedicarboxylate (**8b**) and Preparation of 1,3,4-Oxazole Derivative **17**

Further investigation was done toward compounds **11a**–**11e** and **12a**–**12e** through their reactions with diethyl acetylenedicarboxylate (**8b**). The corresponding oxothiazoles **14a**–**14e** and **15a**–**15e** were obtained and were identified by IR and NMR spectra in addition to HRMS. For example, the structure of compound **14b** was elucidated by ^1^H NMR spectrum *via* the appearance of the aromatic protons as two multiplets at δ_H_ = 7.58–7.27 (for 5H) and at δ_H_ = 6.66–6.22 ppm (6H), whereas the vinyl-proton of the exocyclic double bond resonated as a singlet δ_H_ = 6.78 ppm. A quartet at δ_H_ = 5.22 (*J* = 8.2 Hz, for CH_2_) and as a triplet (3H) at δ_H_ = 1.20 (*J* = 6.9 Hz, CH_3_) appeared to indicate the ethyl ester protons. The benzyl protons are clearly resonated as a double-doublet at δ_H_ = 4.17 ppm (*J* = 14.3, 6.8 Hz). The ^13^C NMR spectrum supported the structure of compound of **14b***via* the appearance of the carbonyl carbon signals at δ_C_ = 150.3 and at 147.5 ppm. The ester carbons and the benzyl carbon signals appeared at δ_C_ = 61.4 (ester-CH_2_), 13.90 (ester-CH_3_), and 45.9 ppm (CH_2_-benzyl).

**Figure 5:**
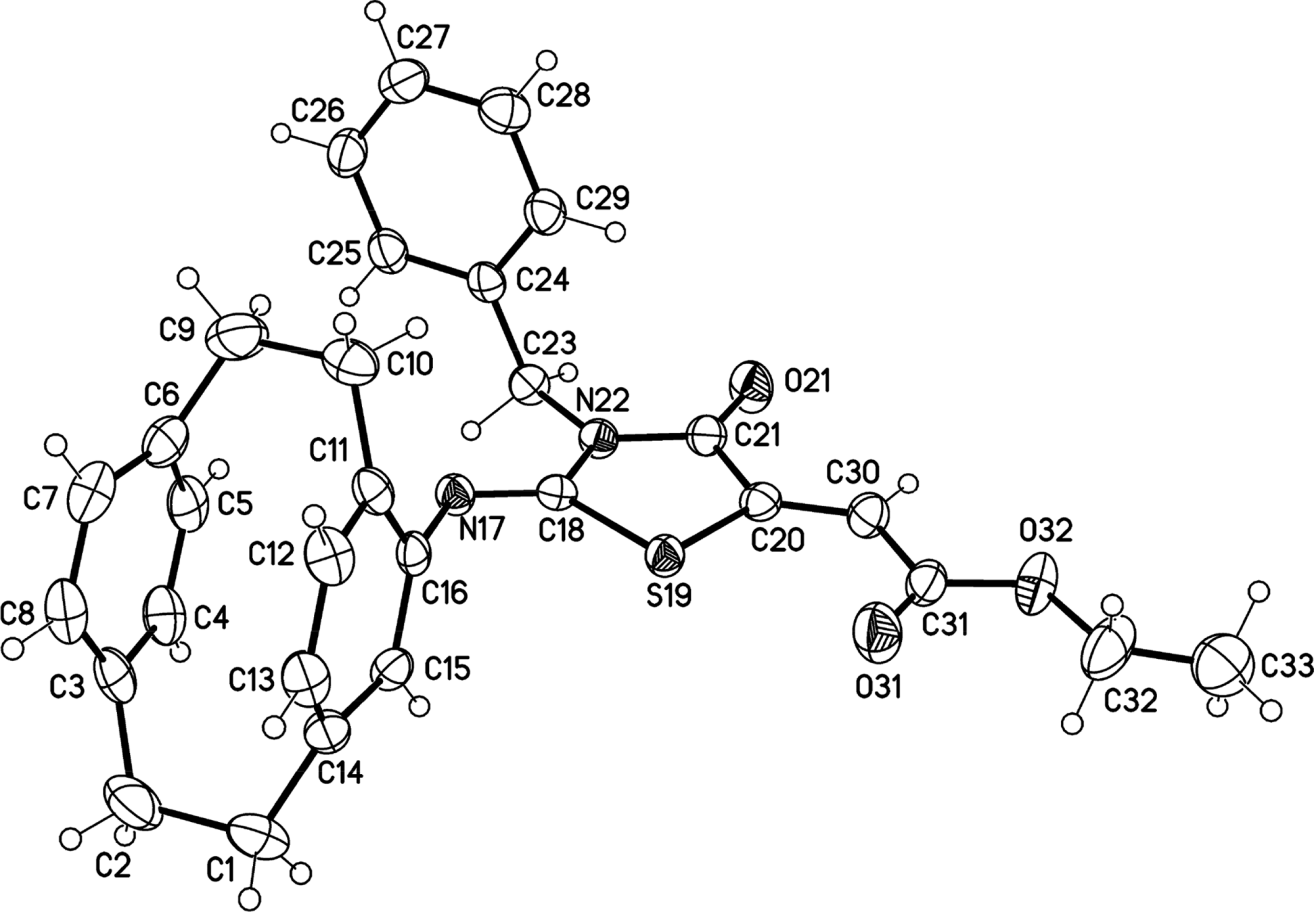
Molecular structure of compound **14b** (minor disordered parts omitted for clarity, displacement parameters are drawn at 50% probability level).

**Figure 6:**
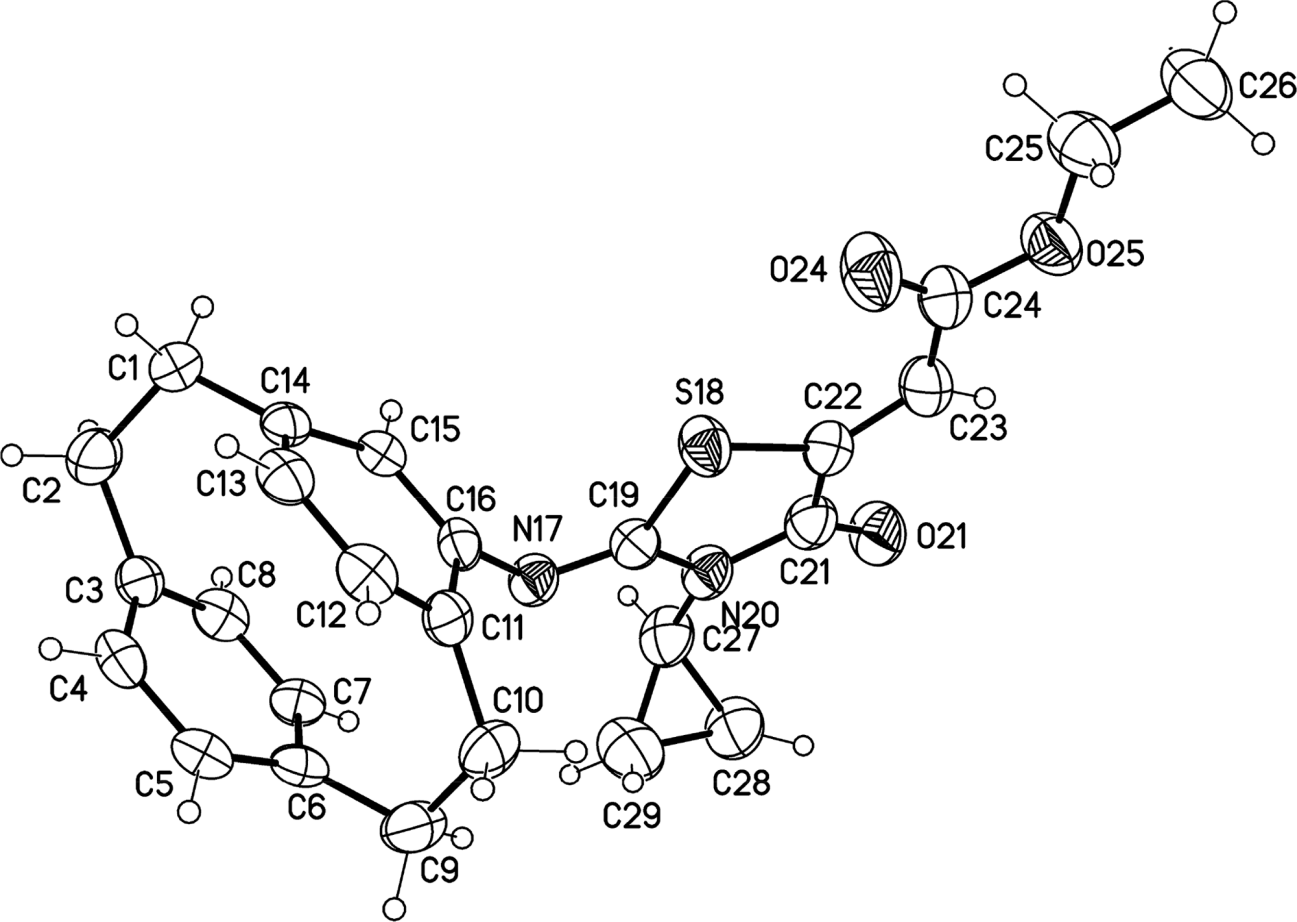
Molecular structure of compound **14e** (displacement parameters are drawn at 50% probability level).

The structure of compound **14b** was totally confirmed by X-ray analysis as shown in Figure 5. X-ray structure analysis also proved the structure of the other thiazole named (*rac*)-ethyl-(*E*)-2-((*E*)-2-(4′-[2.2]paracyclophanylimino)-3-cyclopropyl-4-oxothiazolidin-5-ylidene)acetate (Figure 6).

On the other side, compound **15c** was obtained in 75% yield and it was identified as (*rac*)-ethyl-(*E*)-2-((*E*)-2-(2-(4′-[2.2]paracyclophanylcarbamoyl)-hydrazineylidene)-3-allyl-4-oxothiazolidin-5-ylidene)acetate. The ^1^H NMR spectrum indicated the NH protons as two singlets at δ_H_ = 9.25 (NH-2) and 8.53 ppm (NH-1). The vinyl proton resonated as a singlet at δ_H_ = 6.80. The allyl protons appeared at δ_H_ = 6.08 as ddd (CH-allyl, *J* = 22.4, 10.3, 5.2 Hz), at δ_H_ = 5.12–4.93 as a multiplet for CH_2_-allyl, and at δ_H_ = 4.29 ppm as a doublet (*J* = 5.3 Hz). Finally the ethyl protons appeared, as expected, as a quartet at δ_H_ = 4.18 (CH_2_, *J* = 7.1 Hz), and triplet at δ_H_ = 1.14 ppm (CH_3_, *J* = 7.1 Hz). Three distinguished carbonyl carbon signals in the ^13^C NMR spectrum were present at δ_C_ = 165.3 (CO), 165.1 (CO), and 162.9 ppm. Besides that, the allyl carbons are shown at δ_C_ = 132.8 (=CH), 117.6 (=CH_2_), and at 44.5 ppm (CH_2_−).

In the way to synthesize 1,3,4-oxazole derivative **16**, one example, such as **12e**, was chosen (Scheme 4). The disappearance of the carbonyl and C=S carbons in the IR and ^13^C NMR indicated that cyclization occurred (Scheme 4). The ^1^H NMR spectrum of **16** showed the two NH protons as two singlets at δ_H_ = 9.41 and 8.44 ppm (see the Experimental Section). The allyl protons appeared as a doublet at δ_H_ = 6.76 (*J* = 1.4 Hz), besides two multiplets at δ_H_ = 5.98–5.90 and at δ_H_ = 5.35 and 5.25. According to the ^13^C NMR spectrum of compound **16**, three carbons were distinguished for the allyl carbons at δ_C_ = 46.3 (CH_2_), 115.8 (=CH_2_), and 132.3 ppm (=CH−), respectively.

## 3 Experimental Section

Uncorrected melting points were taken in a Gallenkamp melting point apparatus (Weiss-Gallenkamp, Loughborough, U.K.). The infrared spectra were determined with a Bruker Alpha ATR instrument. The NMR spectra of the title compounds described herein were recorded on a Bruker Avance 400 NMR instrument at 400 MHz for ^1^H NMR and 101 MHz for ^13^C NMR; the references used were the ^1^H and ^13^C peaks of the solvents, *d*_6_-dimethyl sulfoxide ((CD_3_)_2_SO-*d*_6_): 2.50 ppm for ^1^H NMR and 39.4 ppm for ^13^C NMR. For the characterization of centrosymmetric signals, the signal’s median point was chosen; for multiplets, the signal range was given. The following abbreviations were used to describe the proton splitting pattern: d = doublet, t = triplet, m = multiplet, dd = doublet of a doublet. The following abbreviations were used to distinguish between signals: *H*^Ar^ = aromatic-CH, *H*^Pc^ = [2.2]paracyclophane-CH_2_. Signals of the ^13^C NMR spectra were assigned with the help of DEPT90 and DEPT135 and were specified in the following way: + = primary or tertiary carbon atoms (positive DEPT signal), – = secondary carbon atoms (negative DEPT signal), C_q_ = quaternary carbon atoms (no DEPT signal). Mass spectra observed by fast atom bombardment (FAB) experiments were recorded using a Finnigan, MAT 90 (70 eV) instrument. TLC silica plates coated with fluorescence indicator from Merck (silica gel 60 F254, thickness 0.2 mm) were used to purify the crude products; flash chromatography with silica gel 60 (0.040 mm × 0.063 mm, Merck) was used.

### 3.1 General Procedures

Compounds **2**–**5** were prepared according to the literature.^23^

### 3.2 Synthesis of Compound **6**

Isocyanato[2.2]paracyclophane (**5**)^23^ (1.00 g, 4.1 mmol, 1.00 equiv) was fused with benzylamine (5 mL) at 100 °C for 10 h. The reaction mixture was then cooled to room temperature until a precipitate was formed (24 h). The precipitate of **6** was filtered and washed with 150 mL of hexane (three times) and then was dried.

#### (*rac*)-1-(4′-[2.2]Paracyclophanyl))-3-benzylurea (**6**)

*R*_*f*_ = 0.30 (cyclohexane/ethyl acetate, 4:1). Colorless crystals (EtOH), 310 mg (87%). Mp: 150–152 °C. ^1^H NMR (400 MHz, DMSO-*d*_6_): δ_H_ = 7.73 (s, 1H, N*H*^1^), 7.44–7.17 (m, 5H, *H*^Ar^), 6.75 (s, 1H, N*H*^2^), 6.55–6.22 (m, 7H, *H*^Ar^), 4.24 (d, *J* = 6.0 Hz, 2H, C*H*_2_^benzyl^), 3.09–2.81 (m, 7H, *H*^Pc^), 2.67–2.62 ppm (m, 1H, *H*^Pc^). ^13^C NMR (101 MHz, DMSO-*d*_6_): δ_C_ = 158.1 (C_q_, *C*O), 155.1 (C_q_, *C*^Ar^), 140.9 (C_q_, *C*^Ar^), 140.4 (C_q_, *C*^Ar^), 139.9 (C_q_, *C*^Ar^), 138.8 (C_q_, *C*^Ar^), 138.5 (+, *C*H^Ar^), 134.6 (+, *C*H^Ar^), 132.9 (+, *C*H^Ar^), 131.8 (+, *C*H^Ar^), 128.6 (+, *C*H^Ar^), 128.3 (+, *C*H^Ar^), 128.2 (+, *C*H^Ar^), 127.7 (C_q_, *C*^Ar^), 127.1 (+, *C*H^Ar^), 126.7 (+, *C*H^Ar^), 126.5 (+, *C*H^Ar^), 126.0 (+, *C*H^Ar^), 125.2 (+, *C*H^Ar^), 42.9 (−, *C*H_2_^benzyl^), 34.7 (−, *C*H_2_), 34.6 (−, *C*H_2_), 33.1 (−, *C*H_2_), 32.6 ppm (−, *C*H_2_). IR (ATR): *ṽ* = 3340–3210 (br), 3186 (w), 2910 (s), 2856 (m), 1630 cm^–1^ (s). MS (FAB, 3-NBA): *m*/*z* (%) = 357 (60) [M + H]^+^, 356 (30) [M]^+^. HRMS (FAB, 3-NBA, C_24_H_25_N_2_O, [M + H]^+^) calcd, 357.1967; found, 357.1960.

### 3.3 Synthesis of Compound **7a**

Under an argon atmosphere, a mixture of isocyanato[2.2]paracyclophane (**5**)^23^ (5.00 g, 20.1 mmol, 1.00 equiv) was dissolved in 25 mL of hydrazine monohydrate and heated under reflux for 20 h. The reaction mixture was then cooled to room temperature until a precipitate was formed (24 h). Product **7a** was then filtered and washed with 150 mL of hexane (three times) and then dried.

#### (*rac*)-*N*-(4′-[2.2]Paracyclophanyl)hydrazinecarboxamide (**7a**)

*R*_*f*_ = 0.27 (cyclohexane/ethyl acetate, 4:1). Colorless crystals (EtOH), 5 g (89%). Mp: 170–172 °C. ^1^H NMR (400 MHz, DMSO-*d*_6_): δ_H_ = 7.59 (s, 1H, N*H*^2^), 6.88 (s, 1H, N*H*^1^), 6.82 (dd, *J* = 7.7, 1.7 Hz, 1H, *H*^Ar^), 6.62–6.50 (m, 1H, *H*^Ar^), 6.47–6.29 (m, 3H, *H*^Ar^), 6.74 (d, *J* = 1.4 Hz, 1H, *H*^Ar^), 6.15–5.93 (m, 1H, *H*^Ar^), 4.72 (s, 2H, N*H*^2^) 3.29–2.80 (m, 7H, *H*^Pc^), 2.80–2.66 ppm (m, 1H, *H*^Pc^). ^13^C NMR (101 MHz, DMSO-*d*_6_): δ_C_ = 157.3 (C_q_, *C*O), 140.3 (C_q_, *C*^Ar^), 139.3 (C_q_, *C*^Ar^), 139.1 (C_q_, *C*^Ar^), 138.6 (C_q_, *C*^Ar^), 135.3 (+, *C*H^Ar^), 133.6 (+, *C*H^Ar^), 133.3 (+, *C*H^Ar^), 132.4 (+, *C*H^Ar^), 131.8 (+, *C*H^Ar^), 126.9 (+, *C*H^Ar^), 121.9 (+, *C*H^Ar^), 120.7 (C_q_, *C*^Ar^), 35.4 (−, *C*H_2_), 35.1 (−, *C*H_2_), 32.9 (−, *C*H_2_), 32.3 (−, *C*H_2_), 21.0 ppm (+, *C*H_3_). IR (ATR): *ṽ* = 3352–3214 (br), 3196 (w), 2927 (s), 2848 (m), 1632 cm^–1^ (s). MS (FAB, 3-NBA): *m*/*z* (%) = 282 (50) [M + H]^+^, 281 (30) [M]^+^. HRMS (FAB, 3-NBA, C_17_H_20_N_3_O, [M + H]^+^) calcd, 282.1606; found, 282.1603.

### 3.4 Synthesis of Compound **7b**

Under an argon atmosphere, a mixture of isocyanato[2.2]paracyclophane (**5**)^23^ (0.249 g, 1.00 equiv) was added to phenylhydrazine (0.108 g, 1.00 equiv) in 100 mL of toluene and was refluxed for 20 h. The reaction mixture was then cooled to room temperature until a precipitate was formed (24 h). Product **7b** was then filtered and washed with 50 mL of hexane (three times) and then dried.

#### (*rac*)-*N*-(4′-[2.2]Paracyclophanyl)-2-phenylhydrazine-1-carboxamide (**7b**)

*R*_*f*_ = 0.20 (cyclohexane/ethyl acetate, 4:1). Colorless crystals (EtOH), 321 mg (94%). Mp: 147–149 °C. – ^1^H NMR (400 MHz, DMSO-*d*_6_): δ_H_ = 8.36 (s, 1H, N*H*^2^), 7.97 (br, 1H, N*H*^1^), 7.89 (s, 1H, *H*^Ar^), 7.26 (t, *J* = 7.6 Hz, 3H, *H*^Ar^), 6.73 (s, 2H, *H*^Ar^), 6.60 (s, 1H, N*H*^3^), 6.48 (dd, *J* = 7.8, 1.7 Hz, 2H, *H*^Ar^), 6.33 (ddd, *J* = 28.8, 7.6, 2.1 Hz, 4H, *H*^Ar^), 3.20–2.78 (m, 6H, *H*^Pc^), 2.63 ppm (ddd, *J* = 17.0, 10.6, 6.4 Hz, 2H, *H*^Pc^). ^13^C NMR (101 MHz, DMSO-*d*_6_): δ_C_ = 155.8 (C_q_, *C*O), 149.1 (C_q_, *C*^Ar^), 140.7 (C_q_, *C*^Ar^), 139.3 (C_q_, *C*^Ar^), 139.2 (C_q_, *C*^Ar^), 138.6 (C_q_, *C*^Ar^), 132.4 (+, 2 × *C*H^Ar^), 131.4 (+, 2 × *C*H^Ar^), 129.4 (+, 2 × *C*H^Ar^), 128.1 (+, *C*H^Ar^), 127.8 (C_q_, *C*^Ar^), 127.3 (+, *C*H^Ar^), 124.0 (+, *C*H^Ar^), 120.3 (+, *C*H^Ar^), 116.1 (+, 2 × *C*H^Ar^), 36.4 (−, *C*H_2_), 36.1 (−, *C*H_2_), 35.7 (−, *C*H_2_), 32.2 ppm (−, *C*H_2_). IR (ATR): *ṽ* = 3372–3314 (br), 3200 (w), 2927 (s), 2868 (m), 1642 cm^–1^ (s). MS (FAB, 3-NBA): *m*/*z* (%) = 358 (55) [M + H]^+^, 357 (20) [M]^+^. HRMS (FAB, 3-NBA, C_23_H_24_N_3_O, [M + H]^+^) calcd, 358.1919; found, 358.1920.

### 3.5 Synthesis of Compound **9**

A mixture of [2.2]paracyclophanyl hydrazinecarboxamide (**7a**, 0.281 g, 1.00 mmol, 1.00 equiv) and dimethyl acytelenedicarboxylate (**8a**, 0.142 g, 1.00 mmol, 1.00 equiv) in absolute ethanol (40 mL) was refluxed for 4 h (the reaction was monitored by thin-layer chromatography). After removal of the solvent under reduced pressure, the crude product was purified by column chromatography using cyclohexane/EtOAc 10:1 to afford *racemic*-**9**.

#### (*rac*)-Dimethyl (*Z*)-2-(2-(4′-[2.2]paracyclophanyl-carbamoyl)hydrazineylidene)succinate (**9**)

*R*_*f*_ = 0.25 (dichloromethane/methanol, 10:1). Pale yellow crystals (EtOH), 338 mg (80%). Mp: 208–210 °C. ^1^H NMR (400 MHz, DMSO-*d*_6_): δ_H_ = 11.01 (s, 1H, N*H*^2^), 8.45 (s, 1H, N*H*^1^), 6.82 (s, 1H, *H*^Ar^), 6.72–6.60 (m, 1H, *H*^Ar^), 6.55–6.40 (m, 5H, *H*^Ar^), 3.90 (s, 3H, C*H*_3_), 3.75 (s, 3H, C*H*_3_), 3.35–3.20 (m, 5H, *H*^Pc^), 3.05 (s, 2H, C*H*_2_^vinyl^), 2.95–2.64 ppm (m, 3H, *H*^Pc^). ^13^C NMR (101 MHz, DMSO-*d*_6_): δ_C_ = 168.3 (C_q_, *C*O), 164.1 (C_q_, *C*O), 151.5 (C_q_, *C*O), 140.6 (C_q_, *C*=N), 138.7 (C_q_, *C*^Ar^), 138.4 (C_q_, *C*^Ar^), 136.9 (C_q_, *C*^Ar^), 134.8 (C_q_, *C*^Ar^), 133.3 (+, *C*H^Ar^), 132.9 (+, *C*H^Ar^), 132.2 (+, *C*H^Ar^), 131.5 (+, *C*H^Ar^), 129.0 (+, *C*H^Ar^), 127.3 (+, *C*H^Ar^), 127.2 (+, *C*H^Ar^), 125.2 (C_q_, *C*^Ar^), 52.5 (+, *C*H_3_), 52.1 (+, *C*H_3_), 34.6 (−, *C*H_2_), 34.4 (−, *C*H_2_), 32.6 (−, *C*H_2_), 32.1 (−, *C*H_2_), 31.9 ppm (−, *C*H_2_^vinyl^). IR (ATR): *ṽ* = 3300 (w), 3206 (w), 3070 (w), 2920 (w), 2808 (vw), 1640 (w), 1601 (m), 1547 cm^–1^ (s). MS (FAB, 3-NBA): *m*/*z* (%) = 424 (45) [M + H]^+^, 423 (35) [M]^+^. HRMS (FAB, 3-NBA, C_23_H_26_O_5_N_3_, [M + H]^+^) calcd, 424.1872; found, 424.1870.

### 3.6 Synthesis of Compounds **11a**–**11e** and **12a**–**12e**

A mixture of [2.2]paracyclophanehydrazinecarboxamide (**7a**, 1.00 equiv) and the substituted isothiocyanates (**10**, 1.00 equiv) in 60 mL of ethanol was refluxed 80 °C for 4–8 h (the reaction was monitored by thin-layer chromatography). After removal of the solvent under reduced pressure, the crude residue was purified by column chromatography using ethyl acetate/hexane 5:1 to give compounds **11a**–**11e** and **12a**–**12e**.

#### (*rac*)-2′(4′-[2.2]Paracyclophanyl)-16-yridinedin-3-yl)hydrazine-1-carbothioamide (**11a**)

*R*_*f*_ = 0.42 (dichloromethane/methanol, 10:1). Buff crystals (EtOH), 223 mg (60%). Mp: 155–157 °C. ^1^H NMR (400 MHz, DMSO-*d*_6_): δ_H_ = 9.64 (s, 1H, N*H*^1^), 9.05 (s, 1H, N*H*^3^), 7.51–7.30 (m, 2H, *H*^Ar^), 7.21–7.08 (m, 2H, *H*^Ar^), 6.63–6.27 (m, 6H, *H*^Ar^), 6.20–6.06 (m, 1H, *H*^Ar^), 3.12–2.93 (m, 6H, *H*^Pc^), 2.91–2.85 (m, 1H, *H*^Pc^), 2.81–2.64 (m, 1H, *H*^Pc^), 2.35–2.19 ppm (m, 3H, C*H*_3_). ^13^C NMR (100 MHz, DMSO-*d*_6_): δ_C_ = 179.98 (C_q_, *C*S), 140.50 (C_q_, *C*^Ar^), 139.79 (C_q_, *C*^Ar^), 139.22 (C_q_, *C*^Ar^), 137.98 (C_q_, *C*^Ar^), 137.56 (C_q_, *C*^Ar^), 136.11 (C_q_, *C*^Ar^), 135.3 (+, *C*H^Ar^), 134.05 (+, *C*H^Ar^), 134.02 (+, *C*H^Ar^), 133.56 (+, *C*H^Ar^), 133.21 (+, *C*H^Ar^), 132.69 (+, *C*H^Ar^), 130.9 (+, *C*H^Ar^), 130.2 (C_q_, *C*^Ar^), 129.24 (+, 2 × *C*H^Ar^), 124.24 (+, 2 × *C*H^Ar^), 35.2 (−, *C*H_2_), 34.8 (−, *C*H_2_), 34.5 (−, *C*H_2_), 33.7 (−, *C*H_2_), 21.0 ppm (+, *C*H_3_). IR (ATR): *ṽ* = 3296 (w), 3206 (w), 3091 (w), 2925 (w), 1659 (vs), 1594 (m), 1577 (m), 1538 (vs), 1516 (vs), 1494 (vs), 1455 (m), 1436 cm^–1^ (w). MS (FAB, 3-NBA): *m*/*z* (%) = 373 (100) [M + H]^+^, 372 (50) [M]^+^. HRMS (FAB, 3-NBA, C_24_H_25_N_2_^32^S_1_, [M + H]^+^) calcd, 373.1738; found, 373.1740.

#### (*rac*)-1-(4′-[2.2]Paracyclophanyl)-3-benzylthiourea (**11b**)

*R*_*f*_ = 0.40 (dichloromethane/methanol, 10:1). Buff crystals (EtOH), 185 mg (50%). Mp: 165–167 °C. ^1^H NMR (400 MHz, DMSO-*d*_6_): δ_H_ = 9.06 (s, 1H, N*H*^1^), 7.99 (s, 1H, N*H*^3^), 7.34 (d, *J* = 4.4 Hz, 4H, *H*^Ar^), 7.25 (dt, *J* = 5.1, 4.2 Hz, 1H, *H*^Ar^), 6.86 (d, *J* = 5.3 Hz, 1H, *H*^Ar^), 6.53 (dd, *J* = 7.8, 1.7 Hz, 1H, *H*^Ar^), 6.49–6.39 (m, 4H, *H*^Ar^), 6.16 (d, *J* = 1.1 Hz, 1H, *H*^Ar^), 4.72 (ddd, *J* = 19.8, 14.7, 5.7 Hz, 2H, C*H*_2_^benzyl^), 3.16–3.07 (m, 1H, *H*^Pc^), 3.02–2.86 (m, 6H, *H*^Pc^), 2.65 (ddd, *J* = 13.5, 10.1, 5.9 Hz, 1H, *H*^Pc^) ppm. ^13^C NMR (100 MHz, DMSO-*d*_6_): δ_C_ = 181.2 (C_q_, *C*S), 140.8 (C_q_, *C*^Ar^), 139.7 (C_q_, *C*^Ar^), 139.7 (C_q_, *C*^Ar^), 139.2 (C_q_, *C*^Ar^), 137.5 (C_q_, *C*^Ar^), 136.0 (+, *C*H^Ar^), 133.5 (+, *C*H^Ar^), 133.3 (+, *C*H^Ar^), 133.1 (+, *C*H^Ar^), 132.9 (+, *C*H^Ar^), 130.1 (+, *C*H^Ar^), 130.3 (+, *C*H^Ar^), 129.2 (+, *C*H^Ar^), 128.7 (+, 2 × *C*H^Ar^), 128.1 (C_q_, *C*^Ar^), 127.8 (+, *C*H^Ar^), 127.4 (+, *C*H^Ar^), 48.0 (−, *C*H_2_^benzyl^), 35.2 (−, *C*H_2_), 34.8 (−, *C*H_2_), 34.4 (−, *C*H_2_), 33.5 ppm (−, *C*H_2_). IR (ATR) *ṽ* = 3296 (w), 3206 (w), 3091 (w), 2925 (w), 2839 (vw), 1645 (w), 1604 (m), 1557 (s), 1456 (s), 1279 (m), 1129 (vs), 795 (w), 725 (m), 613 (vs), 514 cm^–1^ (w). MS (FAB, 3-NBA): *m*/*z* (%) = 373 (100) [M + H]^+^, 372 (50) [M]^+^. HRMS (FAB, 3-NBA, C_24_H_25_N_2_^32^S_1_, [M + H]^+^) calcd, 373.1738; found, 373.1737.

#### (*rac*)-1-(4′-[2.2]Paracyclophanyl)-3-allylthiourea (**11c**)

*R*_*f*_ = 0.39 (dichloromethane/methanol, 10:1). Buff crystals (EtOH), 167 mg (52%). Mp: 160–162 °C. ^1^H NMR (400 MHz, DMSO-*d*_6_): δ_H_ = 9.04 (s, 1H, N*H*^1^), 7.67 (s, 1H, N*H*^3^), 6.87 (dd, *J* = 7.7, 1.2 Hz, 1H, *H*^Ar^), 6.60–6.40 (m, 5H, *H*^Ar^), 6.15 (d, *J* = 1.4 Hz, 1H, *H*^Ar^), 5.96–5.84 (m, 1H, C*H*^allyl^), 5.26–5.06 (m, 2H, C*H*_2_^allyl^), 4.28–4.00 (m, 2H, C*H*_2_^allyl^), 3.19–3.06 (m, 1H, *H*^Pc^), 3.01–2.87 (m, 6H, *H*^Pc^), 2.70 ppm (ddd, *J* = 13.5, 10.1, 5.9 Hz, 1H, *H*^Pc^). ^13^C NMR (100 MHz, DMSO-*d*_6_): δ_C_ = 180.9 (C_q_, *C*S), 140.8 (C_q_, *C*^Ar^), 139.7 (C_q_, *C*^Ar^), 139.2 (C_q_, *C*^Ar^), 137.5 (C_q_, *C*^Ar^), 136.0 (+, *C*H^Ar^), 135.5 (+, 2 × *C*H^Ar^), 133.4 (+, *C*H^Ar^), 133.1 (+, *C*H^allyl^), 132.8 (+, *C*H^Ar^), 130.9 (+, *C*H^Ar^), 130.2 (+, *C*H^Ar^), 129.2 (C_q_, *C*^Ar^), 116.2 (−, *C*H_2_^allyl^), 46.9 (−, *C*H_2_^allyl^), 35.2 (−, *C*H_2_), 34.8 (−, *C*H_2_), 34.5 (−, *C*H_2_), 33.5 ppm (−, *C*H_2_). IR (ATR): *ṽ* = 3296 (w), 3206 (w), 3091 (w), 2925 (w), 2839 (vw), 1645 (w), 1604 (m), 1557 (s), 1456 cm^–1^ (s). MS (FAB, 3-NBA): *m*/*z* (%) = 323 (100) [M + H]^+^, 322 (55) [M]^+^. HRMS (FAB, 3-NBA, C_20_H_23_N_2_^32^S_1_, [M + H]^+^) calcd, 323.1582; found, 323.1583.

#### (*rac*)-1-(4′-[2.2]Paracyclophanyl)-3-ethylthiourea (**11d**)

*R*_*f*_ = 0.35 (dichloromethane/methanol, 10:1). Buff crystals (MeOH), 179 mg (58%). Mp: 168–170 °C. ^1^H NMR (400 MHz, DMSO-*d*_6_): δ_*H*_ = 8.99 (s, 1H, N*H*^1^), 7.50 (s, 1H, N*H*^3^), 6.86 (dd, *J* = 7.7, 1.7 Hz, 1H, *H*^Ar^), 6.55 (dd, *J* = 7.8, 1.8 Hz, 1H, *H*^Ar^), 6.51–6.40 (m, 4H, *H*^Ar^), 6.12 (d, *J* = 1.5 Hz, 1H, *H*^Ar^), 3.61 (q, 2H, *J* = 7.2 Hz, C*H*_2_^ethyl^), 3.12–3.02 (m, 1H, *H*^Pc^), 3.01–2.88 (m, 6H, *H*^Pc^), 2.70 (ddd, *J* = 13.4, 10.0, 5.9 Hz, 1H, *H*^Pc^), 1.08 ppm (dt, *J* = 13.2, 7.1 Hz, 3H, C*H*_3_^ethyl^). ^13^C NMR (100 MHz, DMSO-*d*_6_): δ_C_ = 180.2 (C_q_, *C*S), 140.9 (C_q_, *C*^Ar^), 139.7 (C_q_, *C*^Ar^), 139.1 (C_q_, *C*^Ar^), 137.4 (C_q_, *C*^Ar^), 136.2 (+, *C*H^Ar^), 135.6 (+, *C*H^Ar^), 133.4 (+, *C*H^Ar^), 133.0 (+, *C*H^Ar^), 132.9 (+, *C*H^Ar^), 130.8 (+, *C*H^Ar^), 129.9 (+, *C*H^Ar^), 129.2 (C_q_, *C*^Ar^), 56.5 (−, C*H*_2_^ethyl^), 35.2 (−, *C*H_2_), 34.8 (−, *C*H_2_), 34.5 (−, *C*H_2_), 33.5 (−, *C*H_2_), 14.9 ppm (+, C*H*_3_^ethyl^). IR (ATR): *ṽ* = 3296 (w), 3206 (w), 3091 (w), 2925 (w), 2839 (vw), 1645 (w), 1604 (m), 1557 (s), 1456 cm^–1^ (s). MS (FAB, 3-NBA): *m*/*z* (%) = 311 (65) [M + H]^+^, 310 (30) [M]^+^. HRMS (FAB, 3-NBA, C_19_H_23_N_2_^32^S_1_, [M + H]^+^) calcd, 311.1582; found, 311.1584.

#### (*rac*)-1-(4′-[2.2]Paracyclophanyl)-3-cyclopropylthiourea (**11e**)

*R*_*f*_ = 0.35 (dichloromethane/methanol, 10:1). Buff crystals (MeOH), 173 mg (54%). Mp: 168–170 °C. ^1^H NMR (400 MHz, DMSO-*d*_6_): δ_H_ = 9.36 (s, 1H, N*H*^1^), 8.32 (s, 1H, N*H*^3^), 6.85 (dd, *J* = 7.8, 1.7 Hz, 2H, *H*^Ar^), 6.57–6.06 (m, 5H, *H*^Ar^), 3.12 (dd, *J* = 20.4, 9.9 Hz, 1H, *H*^Pc^), 3.07–2.81 (m, 7H, *H*^Pc^), 2.75–2.62 (m, 1H, C*H*^cyclo^), 0.75–0.69 (m, 2H, C*H*_2_^cyclo^), 0.65–0.47 ppm (m, 2H, C*H*_2_^cyclo^). ^13^C NMR (100 MHz, DMSO-*d*_6_): δ_C_ = 182.2 (C_q_, *C*S), 140.6 (C_q_, *C*^Ar^), 139.7 (C_q_, *C*^Ar^), 139.2 (C_q_, *C*^Ar^), 135.8 (C_q_, *C*^Ar^), 135.2 (+, *C*H^Ar^), 133.5 (+, *C*H^Ar^), 133.3 (+, *C*H^Ar^), 133.1 (+, *C*H^Ar^), 132.7 (+, *C*H^Ar^), 130.6 (+, *C*H^Ar^), 130.1 (+, *C*H^Ar^), 128.8 (C_q_, *C*^Ar^), 35.2 (−, *C*H_2_), 34.8 (−, *C*H_2_), 34.3 (−, *C*H_2_), 33.7 (−, *C*H_2_), 26.8 (+, C*H*^cyclo.^), 7.1 ppm (−, 2 × C*H*_2_^cyclo^). IR (ATR): *ṽ* = 3296 (w), 3206 (w), 3091 (w), 2925 (w), 2839 (vw), 1645 (w), 1604 (m), 1557 (s), 1456 cm^–1^ (s). MS (FAB, 3-NBA): *m*/*z* (%) = 323 (55) [M + H]^+^, 322 (20) [M]^+^. HRMS (FAB, 3-NBA, C_20_H_23_N_2_^32^S_1_, [M + H]^+^) calcd, 323.1582; found, 323.1583.

#### (*rac*)-*N*-(4′-[2.2]Paracyclophanyl)-2-(*p*-tolylcarbamothioyl)hydrazine-1-carboxamide (**12a**)

*R*_*f*_ = 0.17 (cyclohexane/ethyl acetate, 4:1). Colorless crystals (MeOH), 129 mg (30%). Mp: 190–192 °C. ^1^H NMR (400 MHz, DMSO-*d*_6_): δ_H_ = 9.87 (s, 1H, N*H*^3^), 9.58 (s, 1H, N*H*^2^), 8.52 (s, 1H, N*H*^4^), 7.81 (s, 1H, N*H*^1^), 7.48–7.32 (m, 2H, *H*^Ar^), 7.15 (d, *J* = 8.2 Hz, 3H, *H*^Ar^), 6.90 (dd, *J* = 7.7, 1.5 Hz, 1H, *H*^Ar^), 6.74 (d, *J* = 1.4 Hz, 1H, *H*^Ar^), 6.62–6.25 (m, 4H, *H*^Ar^), 3.15–2.79 (m, 7H, *H*^Pc^), 2.69 (dt, *J* = 13.8, 9.4 Hz, 1H, *H*^Pc^), 2.29 ppm (s, 3H, C*H*_3_). ^13^C NMR (101 MHz, DMSO-*d*_6_): δ_C_ = 180.0 (C_q_, *C*S), 155.0 (C_q_, *C*O), 140.6 (C_q_, *C*^Ar^), 139.3 (C_q_, *C*^Ar^), 139.2 (C_q_, *C*^Ar^), 139.0 (C_q_, *C*^Ar^), 138.2 (C_q_, *C*^Ar^), 137.0 (+, *C*H^Ar^), 135.2 (+, 2 × *C*H^Ar^), 133.5 (+, *C*H^Ar^), 133.2 (+, *C*H^Ar^), 132.4 (+, *C*H^Ar^), 129.6 (+, *C*H^Ar^), 129.2 (C_q_, *C*^Ar^), 129.1 (C_q_, *C*^Ar^), 128.8 (+, *C*H^Ar^), 127.3 (+, *C*H^Ar^), 125.8 (+, *C*H^Ar^), 124.2 (+, *C*H^Ar^), 35.2 (−, *C*H_2_), 35.1 (−, *C*H_2_), 33.4 (−, *C*H_2_), 33.0 (−, *C*H_2_), 21.0 ppm (+, *C*H_3_). IR (ATR): *ṽ* = 3980 (vw), 3954 (vw), 3922 (vw), 3903 (vw), 3870 (vw), 3852 (vw), 3412 (vw), 3352 (vw), 2978 (w), 2925 (w), 2884 (w), 1720 (s), 1696 (m), 1616 (m), 1592 cm^–1^ (vs). MS (FAB, 3-NBA): *m*/*z* (%) = 431 (85) [M + H]^+^, 430 (20) [M]^+^. HRMS (FAB, 3-NBA, C_25_H_27_O_1_N_4_^32^S_1_, [M + H]^+^) calcd, 431.1906; found, 431.1905.

#### (*rac*)-*N*-(4′-[2.2]Paracyclophanyl)-2-(benzylcarbamothioyl)hydrazine-1-carboxamide (**12b**)

*R*_*f*_ = 0.16 (cyclohexane/ethyl acetate, 4:1). Colorless crystals (EtOH), 163 mg (38%). Mp: 186–188 °C. ^1^H NMR (400 MHz, DMSO-*d*_6_): δ_H_ = 9.43 (s, 1H, N*H*^3^), 8.76 (s, 1H, N*H*^2^), 8.44 (s, 1H, N*H*^4^), 7.68 (s, 1H, N*H*^1^), 7.32 (dt, *J* = 19.4, 7.6 Hz, 4H, *H*^Ar^), 7.23 (t, *J* = 7.2 Hz, 1H, *H*^Ar^), 6.85 (dd, *J* = 7.7, 1.2 Hz, 1H, *H*^Ar^), 6.71 (d, *J* = 1.5 Hz, 1H, *H*^Ar^), 6.51–6.31 (m, 5H, *H*^Ar^), 4.80 (ddd, *J* = 40.4, 15.1, 5.8 Hz, 2H, C*H*_2_^benzyl^), 3.26–3.18 (m, 1H, *H*^Pc^), 3.02–2.83 (m, 6H, *H*^Pc^), 2.67 ppm (dt, *J* = 13.7, 8.3 Hz, 1H, *H*^Pc^). ^13^C NMR (101 MHz, DMSO-*d*_6_): δ_C_ = 183.6 (C_q_, *C*S), 155.0 (C_q_, *C*O), 140.6 (C_q_, *C*^Ar^), 139.7 (C_q_, *C*^Ar^), 139.3 (C_q_, *C*^Ar^), 139.0 (C_q_, *C*^Ar^), 138.1 (C_q_, *C*^Ar^), 135.2 (+, *C*H^Ar^), 133.5 (+, *C*H^Ar^), 133.2 (+, *C*H^Ar^), 132.3 (+, *C*H^Ar^), 129.6 (+, *C*H^Ar^), 128.8 (+, *C*H^Ar^), 128.5 (C_q_, *C*^Ar^), 128.4 (C_q_, *C*^Ar^), 127.7 (+, *C*H^Ar^), 127.6 (+, *C*H^Ar^), 127.4 (+, *C*H^Ar^), 127.1 (+, *C*H^Ar^), 125.8 (+, *C*H^Ar^), 47.2 (−, *C*H_2_^benzyl^), 35.2 (−, *C*H_2_), 35.1 (−, *C*H_2_), 33.3 (−, *C*H_2_), 32.9 ppm (−, *C*H_2_). IR (ATR): *ṽ* = 3060 (w), 2983 (w), 2975 (w), 2925 (w), 1715 (s), 1697 (s), 1687 (m), 1628 (m), 1596 cm^–1^ (vs). MS (FAB, 3-NBA): *m*/*z* (%) = 431 (90) [M + H]^+^, 430 (30) [M]^+^. HRMS (FAB, 3-NBA, C_25_H_27_O_1_N_4_^32^S_1_, [M + H]^+^) calcd, 431.1906; found, 431.1904.

#### (*rac*)-*N*-(4′-[2.2]Paracyclophanyl)-2-(allylcarbamothioyl)hydrazine-1-carboxamide (**12c**)

*R*_*f*_ = 0.18 (cyclohexane/ethyl acetate, 4:1). Colorless crystals (EtOH), 148 mg (39%). Mp: 180–182 °C. ^1^H NMR (400 MHz, DMSO-*d*_6_): δ_H_ = 9.35 (s, 1H, N*H*^3^), 8.40 (s, 1H, N*H*^2^), 8.09 (s, 1H, N*H*^4^), 7.66 (s, 1H, N*H*^1^), 6.85 (d, *J* = 7.7 Hz, 1H, *H*^Ar^), 6.71 (d, *J* = 1.2 Hz, 1H, *H*^Ar^), 6.50 (dd, *J* = 7.8, 1.6 Hz, 1H, *H*^Ar^), 6.42–6.35 (m, 3H, *H*^Ar^), 6.31 (dd, *J* = 7.7, 1.6 Hz, 1H, *H*^Ar^), 5.86 (dddt, *J* = 27.4, 17.1, 10.3, 5.1 Hz, 1H, C*H*^allyl^), 5.21–5.02 (m, 2H, C*H*_2_^allyl^), 4.16 (dd, *J* = 20.8, 15.8 Hz, 2H, C*H*2^allyl^), 3.23 (dd, *J* = 10.8, 6.2 Hz, 1H, *H*^Pc^), 3.03–2.86 (m, 6H, *H*^Pc^), 2.72–2.64 ppm (m, 1H, *H*^Pc^). ^13^C NMR (101 MHz, DMSO-*d*_6_): δ_C_ = 182.8 (C_q_, *C*S), 154.9 (C_q_, *C*O), 140.6 (C_q_, *C*^Ar^), 139.3 (C_q_, *C*^Ar^), 139.0 (C_q_, *C*^Ar^), 138.1 (C_q_, *C*^Ar^), 135.4 (+, *C*H^Ar^), 135.2 (+, *C*H^Ar^), 133.5 (+, *C*H^Ar^), 133.2 (+, *C*H^allyl^), 132.3 (+, *C*H^Ar^), 129.5 (+, *C*H^Ar^), 128.8 (+, *C*H^Ar^), 127.3 (C_q_, *C*^Ar^), 125.8 (+, *C*H^Ar^), 115.0 (−, *C*H_2_^allyl^), 46.4 (−, *C*H_2_^allyl^), 35.2 (−, *C*H_2_), 35.1 (−, *C*H_2_), 33.4 (−, *C*H_2_), 32.9 ppm (−, *C*H_2_). IR (ATR): *ṽ* = 3241 (m), 3233 (m), 3109 (m), 2925 (m), 2846 (w), 1646 (w), 1608 (m), 1591 (m), 1560 (vs), 1487 (vs), 1436 (vs) cm^–1^. MS (FAB, 3-NBA): *m*/*z* (%) = 381 (100) [M + H]^+^, 380 (20) [M]^+^. HRMS (FAB, 3-NBA, C_21_H_25_O_1_N_4_^32^S_1_, [M + H]^+^) calcd, 381.1749; found, 381.1750.

#### (*rac*)-*N*-(4′-[2.2]Paracyclophanyl)-2-(ethylcarbamothioyl)hydrazine-1-carboxamide (**12d**)

*R*_*f*_ = 0.15 (cyclohexane/ethyl acetate, 4:1). Colorless crystals (MeOH), 117 mg (32%). Mp: 176–178 °C. ^1^H NMR (400 MHz, DMSO-*d*_6_): δ_H_ = 9.29 (s, 1H, N*H*^3^), 8.37 (s, 1H, N*H*^2^), 8.29 (s, 1H, N*H*^4^), 7.59 (s, 1H, N*H*^1^), 6.85 (dd, *J* = 7.7, 1.3 Hz, 1H, *H*^Ar^), 6.71 (d, *J* = 1.2 Hz, 1H, *H*^Ar^), 6.50 (dd, *J* = 7.8, 1.6 Hz, 1H, *H*^Ar^), 6.42–6.28 (m, 4H, *H*^Ar^), 3.61 (q, 2H, *J* = 7.2 Hz, C*H*_2_-ethyl), 3.27–3.17 (m, 1H, *H*^Pc^), 3.05–2.83 (m, 6H, *H*^Pc^), 2.68 (ddd, *J* = 13.8, 10.0, 7.5 Hz, 1H, *H*^Pc^), 1.12 ppm (t, *J* = 7.1 Hz, 3H, C*H*_3_^ethyl^). ^13^C NMR (101 MHz, DMSO-*d*_6_): δ_C_ = 182.6 (C_q_, *C*S), 155.0 (C_q_, *C*O), 140.6 (C_q_, *C*^Ar^), 139.2 (C_q_, *C*^Ar^), 139.0 (C_q_, *C*^Ar^), 138.0 (C_q_, *C*^Ar^), 135.2 (+, *C*H^Ar^), 133.5 (+, *C*H^Ar^), 133.2 (+, *C*H^Ar^), 132.3 (+, *C*H^Ar^), 129.4 (+, *C*H^Ar^), 128.7 (+, *C*H^Ar^), 127.3 (C_q_, *C*^Ar^), 125.7 (+, *C*H^Ar^), 39.0 (−, *C*H_2_^ethyl^), 35.2 (−, *C*H_2_), 35.0 (−, *C*H_2_), 33.3 (−, *C*H_2_), 32.9 (−, *C*H_2_), 14.9 ppm (+, *C*H_3_^ethyl^). IR (ATR): *ṽ* = 3241 (m), 3233 (m), 3109 (m), 2925 (m), 2846 (w), 1646 (w), 1608 (m), 1591 (m), 1560 (vs), 1487 cm^–1^ (vs). MS (FAB, 3-NBA): *m*/*z* (%) = 369 (50) [M + H]^+^, 368 (20) [M]^+^. HRMS (FAB, 3-NBA, C_20_H_25_O_1_N_3_^32^S_1_, [M + H]^+^) calcd, 369.1749; found, 369.1750.

#### (*rac*)-*N*-(4′-[2.2]Paracyclophanyl)-2-(cyclopropylcarbamothioyl)hydrazine-1-carboxamide (**12e**)

*R*_*f*_ = 0.18 (cyclohexane/ethyl acetate, 4:1). Colorless crystals (DMF/EtOH), 140 mg (37%). Mp: 191–193 °C. ^1^H NMR (400 MHz, DMSO-*d*_6_): δ_H_ = 9.36 (s, 1H, N*H*^3^), 8.31 (s, 1H, N*H*^2^), 8.25 (s, 1H, N*H*^4^), 7.63 (s, 1H, N*H*^1^), 6.97–6.78 (m, 1H, *H*^Ar^), 6.78–6.65 (m, 1H, *H*^Ar^), 6.49 (dt, *J* = 31.4, 15.7 Hz, 1H, *H*^Ar^), 6.42–6.23 (m, 4H, *H*^Ar^), 3.30–3.18 (m, 1H, *H*^Pc^), 3.05–2.83 (m, 6H, *H*^Pc^), 2.70–2.58 (m, 1H, *H*^Pc^), 1.26–1.14 (m, 1H, C*H*^cyclo^), 0.76–0.68 (m, 2H, C*H*_2_^cyclo^), 0.67–0.60 ppm (m, 2H, C*H*_2_^cyclo^). ^13^C NMR (101 MHz, DMSO-*d*_6_): δ_C_ = 184.7 (C_q_, *C*S), 156.2 (C_q_, *C*O), 140.6 (C_q_, *C*^Ar^), 139.3 (C_q_, *C*^Ar^), 139.0 (C_q_, *C*^Ar^), 138.2 (C_q_, *C*^Ar^), 135.2 (+, *C*H^Ar^), 133.5 (+, *C*H^Ar^), 133.2 (+, *C*H^Ar^), 132.3 (+, *C*H^Ar^), 129.4 (+, *C*H^Ar^), 128.8 (+, *C*H^Ar^), 127.2 (C_q_, *C*^Ar^), 125.6 (+, *C*H^Ar^), 35.2 (−, *C*H_2_), 35.1 (−, *C*H_2_), 33.3 (−, *C*H_2_), 32.9 (−, *C*H_2_), 26.8 (+, *C*H^cyclo^), 6.8 ppm (−, 2 × *C*H_2_^cyclo^). IR (ATR): *ṽ* = 3271 (w), 2927 (w), 1751 (m), 1718 (w), 1694 (m), 1656 (vs), 1618 (m), 1601 (m), 1578 (s), 1553 cm^–1^ (s). MS (FAB, 3-NBA): *m*/*z* (%) = 381 (45) [M + H]^+^,380 (20) [M]^+^. HRMS (FAB, 3-NBA, C_21_H_25_O_1_N_3_^32^S_1_, [M + H]^+^) calcd, 381.1749; found, 381.1750.

### 3.7 Synthesis of Thiazoles **14a**–**14e**

A mixture of *N*-substituted [2.2]paracyclophanylthioureas (**11a**–**11e**, 1.00 mmol, 1.00 equiv) and **8b** (0.170 g, 1.00 mmol, 1.00 equiv) in absolute ethanol (40 mL) was refluxed for 3–4 h (the reaction was monitored by thin-layer chromatography). After removal of the solvent under reduced pressure, the crude product was purified by column chromatography using EtOAc/hexane, 5:1 to give compounds **14a**–**14e**.

#### (*rac*)-(*E*)-2-((*E*)-2-(4′-[2.2]Paracyclophanylimino)-4-oxo-3-(*p*-tolyl)thiazolidin-5-ylidene)acetate (**14a**)

*R*_*f*_ = 0.25 (dichloromethane/methanol, 10:1). Yellow crystals (DMF/EtOH), 401 mg (81%). Mp: 238–240 °C. ^1^H NMR (400 MHz, DMSO-*d*_6_): δ_H_ = 8.82 (s, 1H, *H*^Ar^), 8.16 (s, 1H, *H*^Ar^), 7.77 (s, 1H, *H*^vinyl^), 7.30 (d, *J* = 8.4 Hz, 1H, *H*^Ar^), 7.02 (d, *J* = 8.3 Hz, 1H, *H*^Ar^), 6.84 (dd, *J* = 7.7, 1.5 Hz, 1H, *H*^Ar^), 6.75–6.62 (m, 2H, *H*^Ar^), 6.50–6.20 (m, 4H, *H*^Ar^), 3.37 (dd, *J* = 12.4, 10.4 Hz, 2H, C*H*_2_), 3.11–2.76 (m, 6H, *H*^Pc^), 2.75–2.54 (m, 2H, *H*^Pc^), 2.17 (s, 3H, C*H*_3_^phenyl^), 0.98 ppm (t, *J* = 7.0 Hz, 3H, C*H*_3_). ^13^C NMR (101 MHz, DMSO-*d*_6_): δ_C_ = 152.3 (C_q_, *C*O), 151.9 (C_q_, *C*O), 140.0 (C_q_, *C*=N), 138.8 (C_q_, *C*=C), 138.7 (C_q_, *C*^Ar^), 138.6 (C_q_, *C*^Ar^), 138.1 (C_q_, *C*^Ar^), 138.0 (C_q_, *C*^Ar^), 137.4 (C_q_, *C*^Ar^), 134.8 (C_q_, *C*^Ar^), 134.6 (+, *C*H^Ar^), 133.0 (+, *C*H^Ar^), 132.8 (+, *C*H^Ar^), 132.7 (+, *C*H^Ar^), 131.8 (+, *C*H^Ar^), 130.3 (+, *C*H^Ar^), 129.9 (+, *C*H^Ar^), 129.2 (+, *C*H^Ar^), 128.0 (+, *C*H^Ar^), 126.8 (+, *C*H^Ar^), 126.6 (+, *C*H^Ar^), 125.7 (C_q_, *C*^Ar^), 118.0 (+, *C*H^vinyl^), 61.2 (+, *C*H_2_), 34.7 (−, *C*H_2_), 34.5 (−, *C*H_2_), 33.3 (−, *C*H_2_), 33.1 (−, *C*H_2_), 32.6 (+, C*H*_3_^phenyl^), 20.3 ppm (+, C*H*_3_). IR (ATR): *ṽ* = 3296 (w), 3206 (w), 3091 (w), 2925 (w), 2839 (vw), 1645 (w), 1604 (m), 1557 cm^–1^ (s). MS (FAB, 3-NBA): *m*/*z* (%) = 497 (45) [M + H]^+^, 496 (30) [M]^+^. HRMS (FAB, 3-NBA, C_30_H_29_O_3_N_2_^32^S_1_, [M + H]^+^) calcd, 497.1899; found, 497.1891.

#### (*rac*)-(*E*)-2-((*E*)-2-(4′-[2.2]Paracyclophanylimino)-3-benzyl-4-oxothiazolidin-5-ylidene)acetate (**14b**)

*R*_*f*_ = 0.20 (cyclohexane/ethyl acetate, 4:1). Yellow crystals (EtOH), 226 mg (86%). Mp: 230–232 °C. ^1^H NMR (400 MHz, DMSO-*d*_6_): δ_H_ = 7.58–7.27 (m, 5H, *H*^Ar^), 6.78 (s, 1H, *H*^vinyl^), 6.66–6.22 (m, 6H, *H*^Ar^), 5.82 (s, 1H, *H*^Ar^), 5.22 (q, *J* = 8.2 Hz, 2H, C*H*_2_), 4.17 (dd, *J* = 14.3, 6.8 Hz, 2H, C*H*_2_^benzyl^), 3.12–2.59 (m, 8H, *H*^Pc^), 1.20 ppm (t, *J* = 8.0 Hz, 3H, C*H*_3_). ^13^C NMR (101 MHz, DMSO-*d*_6_): δ_C_ = 150.3 (C_q_, *C*O), 147.5 (C_q_, *C*O), 141.4 (C_q_, *C*=N), 139.0 (C_q_, *C*=C), 138.6 (C_q_, *C*^Ar^), 136.0 (C_q_, *C*^Ar^), 134.8 (C_q_, *C*^Ar^), 133.7 (C_q_, *C*^Ar^), 133.1 (C_q_, *C*^Ar^), 132.5 (+, *C*H^Ar^), 132.0 (+, *C*H^Ar^), 131.7 (+, *C*H^Ar^), 130.5 (+, *C*H^Ar^), 129.7 (+, *C*H^Ar^), 129.2 (+, *C*H^Ar^), 128.8 (+, *C*H^Ar^), 128.6 (+, *C*H^Ar^), 127.6 (+, *C*H^Ar^), 127.1 (+, *C*H^Ar^), 126.6 (+, *C*H^Ar^), 122.4 (+, *C*H^Ar^), 121.2 (C_q_, *C*^Ar^), 115.5 (+, *C*H^vinyl^), 61.4 (−, *C*H_2_), 45.9 (−, C*H*_2_^benzyl^), 34.6 (−, *C*H_2_), 34.4 (−, *C*H_2_), 33.3 (−, *C*H_2_), 31.8 (−, *C*H_2_), 13.9 ppm (+, *C*H_3_). IR (ATR): *ṽ* = 3296 (w), 3206 (w), 3091 (w), 2925 (w), 2839 (vw), 1645 (w), 1604 (m), 1557 cm^–1^ (s). MS (FAB, 3-NBA): *m*/*z* (%) = 497 (65) [M + H]^+^, 496 (35) [M]^+^. HRMS (FAB, 3-NBA, C_30_H_29_O_3_N_2_^32^S_1_, [M + H]^+^) calcd, 497.1899; found, 497.1896.

#### (*rac*)-Ethyl-(*E*)-2-((*E*)-2-(4′-[2.2]paracyclophanylimino)-3-allyl-4-oxothiazolidin-5-ylidene)acetate (**14c**)

*R*_*f*_ = 0.17 (cyclohexane/ethyl acetate, 4:1). Yellow crystals (EtOH), 379 mg (85%). Mp: 241–243 °C. ^1^H NMR (400 MHz, DMSO-*d*_6_): δ_H_ = 6.92 (dd, *J* = 7.7, 1.6 Hz, 1H, *H*^Ar^), 6.74 (s, 1H, *H*^vinyl^), 6.61–6.32 (m, 5H, *H*^Ar^), 6.08 (ddd, *J* = 22.4, 10.3, 5.2 Hz, 1H, C*H*^Allyl^), 5.84 (d, *J* = 1.0 Hz, 1H, *H*^Ar^), 5.42–5.27 (m, 2H, C*H*_2_^Allyl^), 4.64 (d, *J* = 5.1 Hz, 2H, C*H*_2_^Allyl^), 4.17 (q, *J* = 7.1 Hz, 2H, C*H*_2_), 3.24–3.12 (m, 1H, *H*^Pc^), 3.08–2.88 (m, 6H, *H*^Pc^), 2.68–2.55 (m, 1H, *H*^Pc^), 1.20 ppm (t, *J* = 7.1 Hz, 3H, C*H*_3_). ^13^C NMR (101 MHz, DMSO-*d*_6_): δ_C_ = 165.2 (C_q_, *C*O), 163.7 (C_q_, *C*O), 147.1 (C_q_, *C*=N), 144.8 (C_q_, *C*=C), 141.5 (C_q_, *C*^Ar^), 140.8 (C_q_, *C*^Ar^), 139.1 (C_q_, *C*^Ar^), 138.6 (C_q_, *C*^Ar^), 134.8 (+, *C*H^Ar^), 133.2 (+, *C*H^Ar^), 132.7 (+, *C*H^Ar^), 132.5 (+, *C*H^Allyl^), 131.7 (+, *C*H^Ar^), 131.5 (+, *C*H^Ar^), 129.6 (+, *C*H^Ar^), 129.3 (+, *C*H^Ar^), 126.7 (C_q_, *C*^Ar^), 117.1 (−, *C*H_2_^Allyl^), 115.2 (+, *C*H^vinyl^), 61.4 (−, *C*H_2_), 44.8 (−, *C*H_2_^Allyl^), 34.7 (−, *C*H_2_), 34.3 (−, *C*H_2_), 33.4 (−, *C*H_2_), 32.1 (−, *C*H_2_), 13.9 ppm (+, *C*H_3_). IR (ATR): *ṽ* = 3421 (vw), 3303 (w), 3063 (w), 2945 (w), 2925 (w), 2851 (w), 1697 (s), 1655 (s), 1602 (vs), 1538 cm^–1^ (m). MS (FAB, 3-NBA): *m*/*z* (%) = 447 (100) [M + H]^+^, 446 (40) [M]^+^. HRMS (FAB, 3-NBA, C_26_H_27_O_3_N_2_^32^S_1_, [M + H]^+^) calcd, 447.1747; found, 447.1737.

#### (*rac*)-Ethyl-(*E*)-2-((*E*)-2-(4′-[2.2]paracyclophanylimino)-3-ethyl-4-oxothiazolidin-5-ylidene)acetate (**14d**)

*R*_***f***_ = 0.14 (cyclohexane/ethyl acetate, 4:1). Yellow crystals (EtOH), 381 mg (88%). Mp: 220–222 °C. ^1^H NMR (400 MHz, DMSO-*d*_6_): δ_H_ = 6.94 (d, *J* = 6.6 Hz, 1H, *H*^Ar^), 6.71 (s, 1H, *H*^vinyl^), 6.60–6.32 (m, 5H, *H*^Ar^), 5.85 (s, 1H, *H*^Ar^), 4.16 (q, *J* = 7.1 Hz, 2H, C*H*_2_), 4.06 (q, *J* = 7.1 Hz, 2H, C*H*_2_^ethyl^), 3.25–3.21 (m, 1H, *H*^Pc^), 3.04–2.90 (m, 6H, *H*^Pc^), 2.69–2.61 (m, 1H, *H*^Pc^), 1.40 (t, *J* = 6.7 Hz, 3H, C*H*_3_^ethyl^), 1.19 ppm (t, *J* = 7.0 Hz, 3H, C*H*_3_). ^13^C NMR (101 MHz, DMSO-*d*_6_): δ_C_ = 165.2 (C_q_, *C*O), 163.8 (C_q_, *C*O), 147.4 (C_q_, *C*=N), 145.0 (C_q_, *C*=C), 141.7 (C_q_, *C*^Ar^), 140.8 (C_q_, *C*^Ar^), 139.1 (C_q_, *C*^Ar^), 138.7 (C_q_, *C*^Ar^), 134.8 (+, *C*H^Ar^), 133.2 (+, *C*H^Ar^), 132.6 (+, 2 × *C*H^Ar^), 131.7 (C_q_, *C*^Ar^), 129.6 (+, *C*H^Ar^), 129.2 (+, *C*H^Ar^), 126.8 (+, *C*H^Ar^), 114.9 (+, *C*H^vinyl^), 61.3 (−, *C*H_2_), 38.0 (−, *C*H_2_^ethyl^), 34.6 (−, *C*H_2_), 34.3 (−, *C*H_2_), 33.5 (−, *C*H_2_), 32.2 (−, *C*H_2_), 13.9 (+, *C*H_3_), 12.5 ppm (+, *C*H_3_^ethyl^). IR (ATR): *ṽ* = 3163 (vw), 3030 (vw), 2929 (w), 2815 (w), 2628 (vw), 1694 (s), 1649 (m), 1606 (vs), 1545 cm^–1^ (s). MS (FAB, 3-NBA): *m*/*z* (%) = 435 (100) [M + H]^+^, 434 (90) [M]^+^. HRMS (FAB, 3-NBA, C_25_H_27_O_3_N_2_^32^S_1_, [M + H]^+^) calcd, 435.1742; found, 435.1743.

#### (*rac*)-Ethyl-(*E*)-2-((*E*)-2-(4′-[2.2]paracyclophanylimino)-3-cyclopropyl-4-oxothiazolidin-5-ylidene)acetate (**14e**)

*R*_*f*_ = 0.55 (cyclohexane/ethyl acetate, 1:1). Yellow crystals (EtOH), 370 mg (83%). Mp: 180–182 °C. ^1^H NMR (400 MHz, DMSO-*d*_6_): δ_H_ = 7.01 (d, *J* = 7.3 Hz, 1H, *H*^Ar^), 6.65 (s, 1H, *H*^vinyl^), 6.60–6.34 (m, 5H, *H*^Ar^), 5.82 (s, 1H, *H*^Ar^), 4.14 (q, *J* = 7.1 Hz, 2H, C*H*_2_), 3.29–3.18 (m, 1H, *H*^Pc^), 3.12–2.91 (m, 7H, *H*^Pc^), 2.70–2.58 (m, 1H, C*H*^cyclo^), 1.17–1.14 (m, 4H, 2 × C*H*_2_^cyclo^), 1.06 ppm (t, *J* = 6.8 Hz, 3H, C*H*_3_). ^13^C NMR (101 MHz, DMSO-*d*_6_): δ_C_ = 165.3 (C_q_, *C*O), 164.4 (C_q_, *C*O), 148.2 (C_q_, *C*=N), 145.5 (C_q_, *C*=C), 142.0 (C_q_, *C*^Ar^), 140.8 (C_q_, *C*^Ar^), 139.2 (C_q_, *C*^Ar^), 138.6 (C_q_, *C*^Ar^), 134.7 (+, *C*H^Ar^), 133.2 (+, *C*H^Ar^), 132.5 (+, *C*H^Ar^), 132.4 (+, *C*H^Ar^), 131.8 (+, *C*H^Ar^), 129.3 (C_q_, *C*^Ar^), 129.2 (+, *C*H^Ar^), 126.8 (+, *C*H^Ar^), 114.4 (+, *C*H^vinyl^), 61.2 (−, *C*H_2_), 34.7 (−, *C*H_2_), 34.3 (−, *C*H_2_), 33.4 (−, *C*H_2_), 32.2 (−, *C*H_2_), 25.6 (+, *C*H^cyclo^), 13.9 (+, *C*H_3_^ethyl^), 6.4 (−, *C*H_2_^cyclo^), 6.3 ppm (−, *C*H_2_^cyclo^). IR (ATR): *ṽ* = 3315 (vw), 3187 (vw), 3013 (w), 2927 (m), 2851 (w), 1714 (s), 1697 (s), 1653 (s), 1606 (vs), 1545 cm^–1^ (s). MS (FAB, 3-NBA): *m*/*z* (%) = 447 (70) [M + H]^+^, 446 (35) [M]^+^. HRMS (FAB, 3-NBA, C_26_H_27_O_3_N_2_^32^S_1_, [M + H]^+^) calcd, 447.1742; found, 447.1739.

### 3.8 Synthesis of Thiazoles **15a**–**15e**

A mixture of *N*-substituted [2.2]paracyclophanylhydrazinecarbothioamides (**12a**–**12e**, 1.00 mmol, 1.00 equiv) and diethyl acetylenedicarboxylate (DEAD) (**8b**, 0.170 g, 1.00 mmol, 1.00 equiv) in absolute ethanol (40 mL) was refluxed for 3–6 h (the reaction was monitored by thin-layer chromatography). After removal of the solvent under reduced pressure, the crude product was purified by column chromatography using EtOAc/hexane, 5:1 to afford **15a**–**15e**.

#### (*rac*)*-*Ethyl-(*E*)-2-((*E*)-2-(2-(4′-[2.2]paracyclophanylcarbamoyl)hydrazineylidene)-4-oxo-3-(*p*-tolyl)thiazolidin-5-ylidene)acetate (**15a**)

*R*_*f*_ = 0.30 (dichloromethane/methanol, 10:1). Yellow crystals (EtOH), 387 mg (70%). Mp: 268–270 °C. ^1^H NMR (400 MHz, DMSO-*d*_6_) δ_H_ = 11.32 (s, 1H, N*H*^2^), 10.75 (s, 1H, N*H*^1^), 7.65–7.40 (m, 4H, *H*^Ar^), 7.30–7.22 (m, 1H, *H*^Ar^), 7.07–6.90 (m, 1H, *H*^Ar^), 6.80 (s, 1H, *H*^vinyl^), 6.75–6.62 (m, 1H, *H*^Ar^), 6.59–6.40 (m, 3H, *H*^Ar^), 3.37 (dd, *J* = 12.4, 10.4 Hz, 2H, C*H*_2_), 3.25–2.70 (m, 8H, *H*^Pc^), 2.18 (s, 3H, C*H*_3_^phenyl^), 1.40 ppm (t, *J* = 7.0 Hz, 3H, C*H*_3_). ^13^C NMR (101 MHz, DMSO-*d*_6_) δ_C_ = 165.4 (C_q_, *C*O), 160.4 (C_q_, *C*O), 152.0 (C_q_, *C*O), 141.0 (C_q_, *C*=N), 140.2 (C_q_, *C*=C), 139.6 (C_q_, *C*^Ar^), 139.4 (C_q_, *C*^Ar^), 139.3 (C_q_, *C*^Ar^), 138.9 (C_q_, *C*^Ar^), 137.7 (C_q_, *C*^Ar^), 135.0 (C_q_, *C*^Ar^), 134.0 (+, *C*H^Ar^), 132.6 (+, *C*H^Ar^), 132.4 (+, *C*H^Ar^), 132.2 (+, *C*H^Ar^), 132.1 (+, *C*H^Ar^), 131.2 (+, *C*H^Ar^), 129.6 (+, *C*H^Ar^), 129.5 (+, *C*H^Ar^), 129.0 (+, *C*H^Ar^), 128.1 (+, *C*H^Ar^), 125.2 (+, *C*H^Ar^), 120.5 (C_q_, *C*^Ar^), 118.0 (+, *C*H^vinyl^), 52.6 (+, *C*H_2_), 34.7 (−, *C*H_2_), 34.5 (−, *C*H_2_), 34.3 (−, *C*H_2_), 34.2 (−, *C*H_2_), 32.5 (+, C*H*_3_^phenyl^), 20.9 ppm (+, C*H*_3_). IR (ATR) *ṽ* = 3296 (w), 3206 (w), 3091 (w), 2925 (w), 2839 (vw), 1645 (w), 1604 (m), 1557 (s), 1456 cm^–1^ (s). MS (FAB, 3-NBA): *m*/*z* (%) = 555 (65) [M + H]^+^, 554 (30) [M]^+^. HRMS (FAB, 3-NBA, C_31_H_31_O_4_N_4_^32^S_1_, [M + H]^+^) calcd, 555.2066; found, 555.2063.

#### (*rac*)-Ethyl-(*E*)-2-((*E*)-2-(2-(4′-[2.2]Paracyclophanylcarbamoyl)hydrazineylidene)-3-benzyl-4-oxothiazolidin-5-ylidene)acetate (**15b**)

*R*_*f*_ = 0.34 (cyclohexane/ethyl acetate, 4:1). Yellow crystals (ethanol), 415 mg (75%). Mp: 280–282 °C. ^1^H NMR (400 MHz, DMSO-*d*_6_) δ_H_ = 10.88 (s, 1H, N*H*^2^), 8.40 (s, 1H, N*H*^1^), 7.58–7.20 (m, 5H, *H*^Ar^), 6.78 (s, 1H, *H*^vinyl^), 6.86–6.20 (m, 7H, *H*^Ar^), 5.19 (q, *J* = 15.2 Hz, 2H, C*H*_2_), 4.18 (dd, *J* = 14.3, 6.8 Hz, 2H, C*H*_2_^benzyl^), 3.12–2.50 (m, 8H, *H*^Pc^), 1.21 ppm (t, *J* = 6.9 Hz, 3H, C*H*_3_). ^13^C NMR (101 MHz, DMSO-*d*_6_): δ_C_ = 165.9 (C_q_, *C*O), 161.5 (C_q_, *C*O), 154.4 (C_q_, *C*O), 140.5 (C_q_, *C*=N), 139.1 (C_q_, *C*=C), 138.6 (C_q_, *C*^Ar^), 137.8 (C_q_, *C*^Ar^), 135.2 (C_q_, *C*^Ar^), 132.5 (C_q_, *C*^Ar^), 132.4 (C_q_, *C*^Ar^), 132.3 (+, *C*H^Ar^), 132.2 (+, *C*H^Ar^), 131.6 (+, *C*H^Ar^), 131.3 (+, *C*H^Ar^), 131.2 (+, *C*H^Ar^), 128.5 (+, 2 × *C*H^Ar^), 128.2 (+, *C*H^Ar^), 127.8 (+, *C*H^Ar^), 127.3 (+, *C*H^Ar^), 127.2 (+, 2 × *C*H^Ar^), 127.0 (C_q_, *C*^Ar^), 116.6 (+, *C*H^vinyl^), 60.4 (−, *C*H_2_), 45.8 (−, C*H*_2_^benzyl^), 34.8 (−, *C*H_2_), 34.6 (−, *C*H_2_), 34.5 (−, *C*H_2_), 34.2 (−, *C*H_2_), 13.8 ppm (+, *C*H_3_). IR (ATR): *ṽ* = 3296 (w), 3206 (w), 3091 (w), 2925 (w), 2839 (vw), 1645 (w), 1604 (m), 1557 (s), 1456 cm^–1^ (s). MS (FAB, 3-NBA): *m*/*z* (%) = 555 (75) [M + H]^+^, 554 (20) [M]^+^. HRMS (FAB, 3-NBA, C_31_H_31_O_4_N_4_^32^S_1_, [M + H]^+^) calcd, 555.2066; found, 555.2063.

#### (*rac*)-Ethyl-(*E*)-2-((*E*)-2-(2-(4′-[2.2]paracyclophanylcarbamoyl)hydrazineylidene)-3-allyl-4-oxothiazolidin-5-ylidene)acetate (**15c**)

*R*_*f*_ = 0.37 (cyclohexane/ethyl acetate, 4:1). Yellow crystals (EtOH), 368 mg (73%). Mp: 291–293 °C. ^1^H NMR (400 MHz, DMSO-*d*_6_): δ_H_ = 9.25 (s, 1H, N*H*^2^), 8.53 (s, 1H, N*H*^1^), 6.80 (s, 1H, *H*^vinyl^), 6.76–6.55 (m, 2H, *H*^Ar^), 6.36–5.89 (m, 4H, *H*^Ar^), 6.08 (ddd, *J* = 22.4, 10.3, 5.2 Hz, 1H, C*H*^allyl^), 5.84 (d, *J* = 1.0 Hz, 1H, *H*^Ar^), 5.12–4.93 (m, 2H, C*H*_2_^allyl^), 4.29 (d, *J* = 5.3 Hz, 2H, C*H*_2_^allyl^), 4.18 (q, *J* = 7.1 Hz, 2H, C*H*_2_), 3.05–2.70 (m, 7H, *H*^Pc^), 2.56 (dd, *J* = 17.6, 8.3 Hz, 1H, *H*^Pc^), 1.14 ppm (t, *J* = 7.1 Hz, 3H, C*H*_3_). ^13^C NMR (101 MHz, DMSO-*d*_6_): δ_C_ = 165.3 (C_q_, *C*O), 165.1 (C_q_, *C*O), 162.9 (C_q_, *C*O), 161.8 (C_q_, *C*=N), 154.5 (C_q_, *C*=C), 153.0 (C_q_, *C*^Ar^), 147.0 (C_q_, *C*^Ar^), 140.6 (C_q_, *C*^Ar^), 140.0 (C_q_, *C*^Ar^), 139.0 (+, *C*H^Ar^), 138.4 (+, *C*H^Ar^), 134.9 (+, *C*H^Ar^), 132.8 (+, *C*H^Allyl^), 132.0 (+, *C*H^Ar^), 130.8 (+, *C*H^Ar^), 128.6 (+, *C*H^Ar^), 127.7 (+, *C*H^Ar^), 126.0 (C_q_, *C*^Ar^), 117.6 (−, *C*H_2_^Allyl^), 116.1 (+, *C*H^vinyl^), 61.5 (−, *C*H_2_), 44.5 (−, *C*H_2_^Allyl^), 34.7 (−, *C*H_2_), 34.5 (−, *C*H_2_), 33.0 (−, *C*H_2_), 32.9 (−, *C*H_2_), 14.0 ppm (+, *C*H_3_). IR (ATR): *ṽ* = 3291 (w), 3285 (w), 3271 (w), 2980 (w), 2966 (w), 2927 (w), 2851 (w), 1742 (m), 1707 (s), 1693 (s), 1660 (vs), 1613 cm^–1^ (s). MS (FAB, 3-NBA): *m*/*z* (%) = 505 (55) [M + H]^+^, 504 (25) [M]^+^. HRMS (FAB, 3-NBA, C_27_H_29_O_4_N_4_^32^S_1_, [M + H]^+^) calcd, 505.1910; found, 505.1906.

#### (*rac*)-Ethyl-(*E*)-2-((*E*)-2-(2-(4′-[2.2]paracyclophanylcarbamoyl)hydrazineylidene)-3-ethyl-4-oxothiazolidin-5-ylidene)acetate (**15d**)

*R*_*f*_ = 0.34 (cyclohexane/ethyl acetate, 4:1). Yellow crystals (EtOH), 374 mg (76%). Mp: 285–287 °C. ^1^H NMR (400 MHz, DMSO-*d*_6_): δ_H_ = 9.46 (s, 1H, N*H*^2^), 8.45 (s, 1H, N*H*^1^), 6.90 (d, *J* = 6.6 Hz, 1H, *H*^Ar^), 6.74 (s, 1H, *H*^vinyl^), 6.45–6.20 (m, 6H, *H*^Ar^), 4.18 (q, *J* = 7.1 Hz, 2H, C*H*_2_), 4.02 (q, *J* = 7.1 Hz, 2H, C*H*_2_^ethyl^), 3.04–2.80 (m, 6H, *H*^Pc^), 2.68–2.65 (m, 1H, *H*^Pc^), 1.25 (t, *J* = 6.7 Hz, 3H, C*H*_3_^ethyl^), 1.15 ppm (t, *J* = 7.0 Hz, 3H, C*H*_3_). ^13^C NMR (101 MHz, DMSO-*d*_6_): δ_C_ = 164.8 (C_q_, *C*O), 161.3 (C_q_, *C*O), 153.9 (C_q_, *C*O), 146.5 (C_q_, *C*=N), 140.1 (C_q_, *C*=C), 139.5 (C_q_, *C*^Ar^), 138.5 (C_q_, *C*^Ar^), 137.9 (C_q_, *C*^Ar^), 136.6 (C_q_, *C*^Ar^), 134.3 (+, *C*H^Ar^), 132.3 (+, *C*H^Ar^), 131.5 (+, 2 × *C*H^Ar^), 130.2 (C_q_, *C*^Ar^), 128.1 (+, *C*H^Ar^), 127.1 (+, *C*H^Ar^), 125.4 (+, *C*H^Ar^), 115.6 (+, *C*H^vinyl^), 61.0 (−, *C*H_2_), 40.7 (−, *C*H_2_^ethyl^), 34.2 (−, *C*H_2_), 34.0 (−, *C*H_2_), 32.5 (−, *C*H_2_), 32.4 (−, *C*H_2_), 14.0 (+, *C*H_3_), 13.4 ppm (+, *C*H_3_^ethyl^). IR (ATR) *v* = 3163 (vw), 3030 (vw), 2929 (w), 2815 (w), 2628 (vw), 1694 (s), 1649 (m), 1606 (vs), 1545 cm^–1^ (s). MS (FAB, 3-NBA): *m*/*z* (%) = 493 (100) [M + H]^+^, 492 (30) [M]^+^. HRMS (FAB, 3-NBA, C_26_H_29_O_4_N_4_^32^S_1_, [M + H]^+^) calcd, 493.1910; found, 493.1905.

#### (*rac*)-Ethyl-(*E*)-2-((*E*)-2-(4′-[2.2]paracyclophanyl-carbamoyl)hydrazineylidene)-3-cyclopropyl-4-oxothiazolidin-5-ylidene)acetate (**15e**)

*R*_*f*_ = 0.37 (cyclohexane/ethyl acetate, 1:1). Yellow crystals (EtOH), 363 mg (72%). Mp: 260–262 °C. ^1^H NMR (400 MHz, DMSO-*d*_6_): δ_H_ = 9.36 (s, 1H, N*H*^2^), 8.43 (s, 1H, N*H*^1^), 6.87 (s, 1H, *H*^vinyl^), 6.80–6.70 (m, 1H, *H*^Ar^), 6.49 (d, *J* = 7.8 Hz, 2H, *H*^Ar^), 6.45–6.27 (m, 4H, *H*^Ar^), 4.29 (q, *J* = 6.9 Hz, 2H, C*H*_2_), 3.31–3.20 (m, 1H, *H*^Pc^), 3.09–2.80 (m, 7H, *H*^Pc^), 2.72–2.55 (m, 1H, C*H*^cyclo^), 1.29 (t, *J* = 7.0 Hz, 3H, C*H*_3_), 1.03–0.86 (m, 2H, C*H*_2_^cyclo^), 0.79–0.66 ppm (m, 2H, C*H*_2_^cyclo^). ^13^C NMR (101 MHz, DMSO-*d*_6_): δ_C_ = 165.2 (C_q_, *C*O), 161.5 (C_q_, *C*O), 152.8 (C_q_, *C*O), 145.8 (C_q_, *C*=N), 140.0 (C_q_, *C*=C), 138.9 (C_q_, *C*^Ar^), 138.7 (C_q_, *C*^Ar^), 138.5 (+, *C*H^Ar^), 137.5 (C_q_, *C*^Ar^), 136.9 (C_q_, *C*^Ar^), 134.9 (+, *C*H^Ar^), 132.7 (+, *C*H^Ar^), 132.1 (+, *C*H^Ar^), 131.6 (+, *C*H^Ar^), 128.7 (C_q_, *C*^Ar^), 127.9 (+, *C*H^Ar^), 126.2 (+, *C*H^Ar^), 115.7 (+, *C*H^vinyl^), 61.5 (−, *C*H_2_), 34.7 (−, *C*H_2_), 34.5 (−, *C*H_2_), 34.4 (−, *C*H_2_), 34.1 (−, *C*H_2_), 33.1 (+, *C*H^cyclo^), 14.0 (+, *C*H_3_^ethyl^), 8.1 ppm (−, 2 × *C*H_2_^cyclo^). IR (ATR): *ṽ* = 3315 (vw), 3187 (vw), 3013 (w), 2927 (m), 2851 (w), 1714 (s), 1697 (s), 1653 (s), 1606 (vs), 1545 (s), 1436 cm^–1^ (m). MS (FAB, 3-NBA): *m*/*z* (%) = 505 (90) [M + H]^+^, 504 (25) [M]^+^. HRMS (FAB, 3-NBA, C_27_H_29_O_4_N_4_^32^S_1_, [M + H]^+^) calcd, 505.1910; found, 505.1905.

### 3.9 Synthesis of Compound **16**

A stirring mixture of *N*-allyl [2.2]paracyclophanylhydrazinecarbothioamides (**12e**, 0.380 g, 1.00 mmol, 1.00 equiv) and 10 mL of sodium hydroxide (1.00 mmol, as a 2 N solution) dissolved in 40 mL of ethanol was refluxed for 3 h. After cooling, the solution was acidified with 10 mL of hydrochloric acid (6 M) and the formed precipitate was filtered.

#### *N*^2^-(4′-[2.2]Paracyclophanyl)-*N*^5^-allyl-1,3,4-oxadiazole-2,5-diamine (**16**)

*R*_*f*_ = 0.46 (dichloromethane/methanol, 10:1), colorless crystals (CHCl_3_/EtOH), 207 mg (60%). Mp: 206–208 °C. ^1^H NMR (400 MHz, DMSO-*d*_6_): δ_H_ = 9.41 (s, 1H, N*H*), 8.44 (s, 1H, N*H*), 7.72 (s, 1H, *H*^Ar^), 6.91 (dd, *J* = 7.7, 1.3 Hz, 1H, *H*^Ar^), 6.91 (dd, *J* = 7.7, 1.3 Hz, 1H, *H*^Ar^), 6.76 (d, *J* = 1.4 Hz, 1H, C*H*^allyl^), 6.56–6.35 (m, 4H, *H*^Ar^), 5.98–5.90 (m, 2H, C*H*_2_^allyl^), 5.75–5.25 (m, 2H, C*H*_2_^allyl^), 3.10–2.89 ppm (m, 8H, *H*^Pc^). ^13^C NMR (101 MHz, DMSO-*d*_6_): δ_C_ = 155.0 (C_q_, *C*^Ar^), 140.6 (C_q_, *C*^Ar^), 139.7 (C_q_, *C*^Ar^), 139.3 (C_q_, *C*^Ar^), 139.0 (C_q_, *C*^Ar^), 138.1 (C_q_, *C*^Ar^), 135.4 (+, *C*H^Ar^), 135.2 (+, *C*H^Ar^), 133.5 (+, *C*H^Ar^), 133.2 (+, *C*H^Ar^), 132.3 (+, *C*H^allyl^), 129.5 (+, *C*H^Ar^), 128.8 (+, *C*H^Ar^), 127.3 (+, *C*H^Ar^), 125.8 (C_q_, *C*^Ar^), 115.8 (−, *C*H_2_^allyl^), 46.3 (−, *C*H_2_^allyl^), 35.5 (−, *C*H_2_), 35.2 (−, *C*H_2_), 33.4 (−, *C*H_2_), 32.9 ppm (−, *C*H_2_). IR (ATR) *ṽ* = 3214 (w), 3156 (w), 3013 (w), 2946 (m), 2925 (m), 2888 (m), 2856 (w), 1717 (w), 1653 (vs), 1623 (vs), 1595 cm^–1^ (s). MS (FAB, 3-NBA): *m*/*z* (%) = 347 (100) [M + H]^+^, 346 (50) [M]^+^. HRMS (FAB, 3-NBA, C_21_H_23_O_1_N_4_, [M + H]^+^) calcd, 347.1872; found, 347.1870.

### 3.10 Crystal Structure Determinations

The single-crystal X-ray diffraction study were carried out on a Bruker D8 Venture diffractometer with a PhotonII detector at 123(2) K or 173(2) K using Cu–Kα radiation (λ = 1.54178 Å). Dual space/intrinsic methods^25^ were used for structure solution, and refinement was carried out using SHELXL-2014 (full-matrix least-squares on *F*^2^).^26^ Hydrogen atoms were localized by difference electron density determination and refined using a riding model (H(N) free). Semiempirical absorption corrections were applied. For **14b**, an extinction correction was applied. In **14b**, the ethyl moiety is disordered (see the cif files for details). **14e** was refined as a twin with two domains.

**9**: Yellow crystals, C_23_H_25_N_3_O_5_, *M*_r_ = 423.46, crystal size 0.16 × 0.12 × 0.04 mm^3^, monoclinic, space group *P*2_1_/*n* (no. 14), *a* = 13.3388(5) Å, *b* = 8.1948(3) Å, *c* = 19.9695(8) Å, β = 106.989(2)°, *V* = 2087.58(14) Å^3^, *Z* = 4, ρ = 1.347 Mg/m^–3^, μ(Cu–K_α_) = 0.79 mm^–1^, *F*(000) = 896, *T* = 123 K, 2θ_max_ = 144.6°, 32299 reflections, of which 4117 were independent (*R*_int_ = 0.035), 288 parameters, 2 restraints, *R*_1_ = 0.071 (for 3572*I* > 2σ(*I*)), *wR*_2_ = 0.213 (all data), *S* = 1.05, largest diff. peak/hole = 1.00/–0.20 e Å^–3^.

**11d**: Colorless crystals, C_19_H_22_N_2_S, *M*_r_ = 310.44, crystal size 0.16 × 0.06 × 0.04 mm^3^, triclinic, space group *P*-1 (no. 2), *a* = 11.3925(3) Å, *b* = 12.0321(3) Å, *c* = 13.4654(4) Å, α = 88.526(1)°, β = 65.296(1)°, γ = 76.086(1)°, *V* = 1621.79(8) Å^3^, *Z* = 4, ρ = 1.271 Mg/m^–3^, μ(Cu–K_α_) = 1.74 mm^–1^, *F*(000) = 664, *T* = 123 K, 2θ_max_ = 144.8°, 30263 reflections, of which 6380 were independent (*R*_int_ = 0.029), 409 parameters, 4 restraints, *R*_1_ = 0.038 (for 5802*I* > 2σ(*I*)), *wR*_2_ = 0.107 (all data), *S* = 1.05, largest diff. peak/hole = 0.41/–0.27 e Å^–3^.

**14b**: Yellow crystals, C_30_H_28_N_2_O_3_S, *M*_r_ = 496.60, crystal size 0.16 × 0.04 × 0.02 mm^3^, monoclinic, space group *P*2_1_/*c* (no. 14), *a* = 10.0473(4) Å, *b* = 34.0461(14) Å, *c* = 7.5093(3) Å, β = 100.286(2)°, *V* = 2527.43(18) Å^3^, *Z* = 4, ρ = 1.305 Mg/m^–3^, μ(Cu–K_α_) = 1.42 mm^–1^, *F*(000) = 1048, *T* = 173 K, 2θ_max_ = 144.6°, 18990 reflections, of which 4720 were independent (*R*_int_ = 0.053), 325 parameters, 2 restraints, *R*_1_ = 0.045 (for 4229*I* > 2σ(*I*)), *wR*_2_ = 0.122 (all data), *S* = 1.04, largest diff. peak/hole = 0.51/–0.51 e Å^–3^.

**14e**: Yellow crystals, C_26_H_26_N_2_O_3_S, *M*_r_ = 446.55, crystal size 0.21 × 0.15 × 0.03 mm^3^, monoclinic, space group *P*2_1_/*c* (no. 14), *a* = 24.6864(10) Å, *b* = 7.8388(3) Å, *c* = 11.4055(5) Å, β = 91.995(1)°, *V* = 2205.76(16) Å^3^, *Z* = 4, ρ = 1.345 Mg/m^–3^, μ(Cu–K_α_) = 1.56 mm^–1^, *F*(000) = 944, *T* = 173 K, 2θ_max_ = 144.6°, 16496 reflections, of which 4306 were independent (*R*_int_ = 0.043), 290 parameters, *R*_1_ = 0.096 (for 3868*I* > 2σ(*I*)), *wR*_2_ = 0.278 (all data), *S* = 1.04, largest diff. peak/hole = 1.48/–0.52 e Å^–3^.

CCDC-2128196 (**9**), CCDC-2128197 (**11d**), CCDC-2128199 (**14b**), and CCDC-2128199 (**14e**) contain the supplementary crystallographic data for this paper. These data can be obtained free of charge from The Cambridge Crystallographic Data Centre via www.ccdc.cam.ac.uk/data_request/cif

## 4 Conclusion

In the current study, a novel series assembly of thio(ureas), semicarbazides, thiosemicarbazides, thiazoles, and oxadiazole derived from [2.2]paracyclophane were effectively synthesized. Therefore, it would be potentially applied to the symmetrical disubstituted PC. We are encouraging to synthesize numerous new heterocycles derived from [2.2]paracyclophanes aiming to increase attention on that important asymmetric molecule toward biological activity. Previous reports have dealt with effective biological activities resulting from conjugation between paracyclophane and heterocycle molecules. That might led to the discovery of promising novel hybrids of interesting heterocyclic/paracyclophanes as a starting point in medicinal chemistry art that warrants further research and development as potential biological active candidates.

## Notes

CCDC-2128196 (**9**), CCDC-2128197 (**11d**), CCDC-2128199 (**14b**), and CCDC-2128199 (**14e**) contain the supplementary crystallographic data for this paper. These data can be obtained free of charge from The Cambridge Crystallographic Data Centre via www.ccdc.cam.ac.uk/data_request/cif.
